# Isothermal real-time RT-RPA for Machupo virus detection: Field-adaptable sensitivity comparable with laboratory PCR

**DOI:** 10.1371/journal.pone.0340488

**Published:** 2026-01-12

**Authors:** Marina A. Kapitonova, Anna V. Shabalina, Igor S. Sukhikh, Artemiy A. Volkov, Vladimir G. Dedkov, Anna S. Dolgova

**Affiliations:** 1 Laboratory of Pathogen Molecular Genetics, Saint Petersburg Pasteur Institute, Saint Petersburg, Russia; 2 Martsinovsky Institute of Medical Parasitology, Tropical and Vector Borne Diseases, First Moscow State Medical University (Sechenov University), Moscow, Russia; University of Florida Tropical Research and Education Center, UNITED STATES OF AMERICA

## Abstract

Isothermal nucleic acid amplification methods, such as recombinase polymerase amplification (RPA), are becoming increasingly vital as diagnostic platforms for neglected tropical diseases by enabling rapid and accurate detection in under-resourced regions. We developed a real-time RT-RPA assay for Machupo virus (MACV), the causative agent of Bolivian hemorrhagic fever, and directly compared it to a real-time RT-PCR assay targeting the same viral sequence fragment. The methods were evaluated across critical parameters: limit of detection (LOD), tolerance to single-nucleotide substitutions, multiplexing capability, and adaptability to multiple MACV genetic variants. The LOD was identical for both assays: 5 × 10^3^ copies/ml of armored RNA particles. They differed in terms of input RNA (copies/reaction): 100 (PCR) versus 20 (RPA). The real-time RT-RPA assay was further validated on a portable device, demonstrating its potential for field-deployability for point-of-care applications.

## Introduction

Machupo virus (*Mammarenavirus machupoense*, MACV), a member of the *Arenaviridae* family, causes Bolivian hemorrhagic fever (BHF), a severe zoonotic disease endemic to rural Bolivia. First identified during a deadly 1963 outbreak with a 30% case fatality rate [1], MACV persists in nature through its rodent reservoir (*Calomys callosus*), with human infections occurring via aerosolized excreta or direct contact. BHF is classified as a neglected tropical disease (NTD). Like other hemorrhagic fevers caused by arenaviruses, it highlights the critical convergence of zoonotic spillover, limited healthcare access, and diagnostic gaps in resource-poor regions [2]. Diagnosis of NTDs such as BHF presents systemic challenges: inadequate laboratory infrastructure; expensive equipment; and shortages of trained personnel in endemic areas.

Conventional MACV detection methods, serology (IgM/IgG ELISA) [3] and lab-bound reverse-transcription PCR (RT-PCR) [4], cause critical delays in outbreak response in low-resource conditions. Therefore, the development and implementation of novel, rapid, and accurate point-of-care testing (POCT) methods becomes imperative [5]. A key strategy involves isothermal nucleic acid amplification techniques, which enable rapid target detection using portable devices. For instance, loop-mediated isothermal amplification (LAMP) has been successfully deployed for field identification of various bacterial [6,7], parasitic [8,9] and viral.

NTD pathogens. These include dengue [10], Zika [11], Lassa [12], Ebola [13], Marburg, Rift Valley fever, yellow fever, Congo Basin mpox, West African mpox, and variola viruses [14]. Another isothermal nucleic acid amplification method, rolling circle amplification (RCA), has been applied in diagnosis of the NTD black-grain eumycetoma by detecting six potential causative agents [15]. This method has also been utilized for detecting type-specific bovine papillomavirus [16], H5N1 influenza virus [17], as well as the Ebola, dengue, and Zika viruses [18]. Nucleic acid sequence-based amplification (NASBA) has demonstrated high efficacy in detecting diverse viral pathogens, including SARS-CoV-2 [19], rotavirus A [20], Zika virus [21], norovirus [22], and hepatitis B [23].

Among isothermal amplification methods, recombinase polymerase amplification (RPA) has gained significant traction in NTD diagnostics, demonstrating robust performance across diverse pathogens. It has been successfully deployed for parasitic targets [24–28], while equally enabling sensitive detection of high-consequence viruses, such as Crimean-Congo hemorrhagic fever virus (CCHFV) [29], severe fever with thrombocytopenia syndrome virus (SFTSV) [30], monkeypox virus [31], dengue virus [32], and Ebola virus [33]. However, despite the proven utility of isothermal methods for diverse NTD pathogen detection, no field-deployable molecular diagnostics exist for New World arenaviruses, particularly Machupo virus, which causes severe hemorrhagic fevers with pandemic potential. This critical gap leaves endemic regions without rapid tools for outbreak containment. To address this, we developed two methods: a real-time RT-RPA assay targeting a conserved region of the MACV RNA-dependent RNA polymerase gene; and a real-time RT-PCR assay for the same target. Here, we conducted a direct, head-to-head methodological comparison of isothermal RPA and conventional PCR methods. We analyzed sensitivity, cost, the influence of several single-nucleotide mutations on performance, and operational feasibility using armored RNA standards. Armored RNA standards or armored RNA particles (ARPs) encapsulate the target RNA within a protective bacteriophage coat, closely mimicking the structure and extraction challenges posed by authentic viral particles, thereby providing a rigorous and biologically relevant standard for assay. Due to the BSL-4 classification of Machupo virus, which excludes its use in our laboratory setting, ARPs provide a safe and highly reliable model system.

Furthermore, this comparative study addresses an important question in contemporary diagnostics: Can rapid isothermal technologies like RPA realistically replace the gold-standard, PCR? Despite its proven reliability, PCR can be less useful in time-sensitive outbreak scenarios where infrastructure is limited. By rigorously evaluating both assays under identical conditions, we provide direct comparative evidence for transitioning toward field-deployable platforms without compromising sensitivity. This work establishes the first portable, sequence-confirmed diagnostic for MACV while offering a transferable framework for arenavirus detection in resource-constrained settings.

## Materials and methods

All primers for real-time PCR and RPA assays, along with fluorescent probes for RPA (Tables 1, S1 Table in S1 File), were commercially synthesized by DNA-Synthesis (Moscow, Russia). Fluorescent probes for real-time PCR were synthesized by Genterra (Moscow, Russia). Probes for RPA contained a site-specific tetrahydrofuran (THF) residue, introduced as a custom modification by DNA-Synthesis (Moscow, Russia) using dSpacer CE Phosphoramidite (5’-O-dimethoxytrityl-1’,2’-dideoxyribose-3’-[(2-cyanoethyl)-(N,N-diisopropyl)]-phosphoramidite, Cambio, UK). Lyophilized primers were stored at room temperature, while resuspended primers and probes were stored at −20°C. All graphical visualizations, including kinetic plots, heatmaps, and standard curves were generated using MagicPlot 3.0.1 (MagicPlot Systems, Russia).

**Table 1:** Target sequences and optimized primer/probe sequences (PCR/RPA). Nucleotide substitutions relative to MACV are bold.

Name	Sequence (5’ → 3’)
MACV	CATCAACCATCACGTTGCTTAACGAGATTCTAACACCCTCGAAGTTAGAGACAACATACCCCATTGCGATATCCTCCACAAACATAGCTCTGGAAACATTTTGATTCTTAACCATTAAGGCAAAGTCAGTCAATTC
MACV_2	CATCAACCATCA**T**GTTGCTTAACGAGATTCTAACACCCTCGAAGTTAGAGACAACATACCCCATTGCGATATCCTCCACAAACATAGCTCTGGAAACATTTTGATTCTTAACCATTAAGGCAAA**A**TCAGTCAATTC
MACV_10	CATCAACCA**C**CACG**C**TGCTTAA**T**GAGATTCT**G**ACACCCTCGAAGTT**G**GAGACAACATA**A**CCCATTGCGATATCCTCCACAAACATAGCTCTGG**T**AACATT**G**TGATT**T**TTAACCATTAAGGCAAAGTCAGT**T**AATTC
MACV_14	CATCAACCA**C**CACGTTGCT**C**AA**T**GAGATTCT**G**ACACC**T**TC**A**AAGTT**G**GAGACAACATA**A**CCCATTGC**A**ATATCCTCCACAAACATAGC**C**CTGGAAACATTTTGA**C**T**T**TTAACCAT**C**AAGGCAAAGTCAGTCA**G**TTC
PCR_F	TCAACCATCA**Y**GTTGCTTAACG
PCR_F.v2	TCAACCA**C**CACGTTGCT**C**AA**T**G
PCR_R	ATTGACTGA**Y**TTTGCCTTAATGGT
PCR_R.v2	A**C**TGACTGACTTTGCCTT**G**ATGGT
PCR_prb	[R6G] ACCCTCGAAGTTAGAGACAACATACCGAG [BHQ1]
PCR_prb.v2	[R6G] ACC**T**TC**A**AAGTT**G**GAGACAACATA**A**CGAG [BHQ1]
RPA_1F	GAGATTCTAACACCCTCGAAGTTAGAGACAAC
RPA_1F.gtag	GAGATTCT**G**ACACC**T**TC**A**AAGTT**G**GAGACAAC
RPA_1F.2g	GAGATTCTAACACCCTCGAAGTT**G**GAGACAAC
RPA_4R	GAATTGACTGACTTTGCCTTAATGGTTAAGAAT
RPA_4R.gctc	GAA**C**TGACTGACTTTGCCTT**G**ATGGTTAA**A**A**G**T
RPA_4R.2t	GAATTGACTGACTTTGCCTTAATGGTTAA**A**AAT
RPA_2prb	CGAAGTTAGAGACAACATACCCCATTGCGA [dT-FAM] [THF] [dT-BHQ1] CCTCCACAAACATAG [Spacer C3]
RPA_2prb.ga	CGAAGTT**G**GAGACAACATA**A**CCCATTGCGA [dT-FAM] [THF] [dT-BHQ1] CCTCCACAAACATAG [Spacer C3]

### Positive control plasmid construction

MACV target sequences (wild-type and variants) were synthesized *de novo* via PCR assembly using overlapping oligonucleotides, adapted from Dolgova and Stukolova [34]. PCR was conducted using an outer pair of primers (M_1, M_4, 30 pmol) and inner primers (M_2, M_3, 1 pmol) with Phusion High-Fidelity DNA Polymerase (NEB, USA). The thermal cycling program was: 98°C for 30 s; 27 cycles (98°C for 10 s, 62°C for 30 s, 72°C for 10 s); and 72°C for 5 min. Products were purified using the DNA Clean & Concentrator Kit (Zymo Research, USA), A-tailed using Taq DNA Polymerase (Evrogen, Russia), and ligated into pGEM-T (Promega, USA). Plasmids were transformed into NEB Turbo *E. coli* (NEB, USA) and sequence verified.

Variant sequences (MACV_2, MACV_10, MACV_14) were synthesized identically using mutation-specific oligonucleotides (M_2_1–2, M_10_1–4, M_14_1–4). Linear DNA controls were generated by PCR amplification of plasmid inserts (2.3 kb target with flanking regions), purified using AMPure XP beads (Beckman Coulter, USA), and quantified via the NanoDrop One device (Thermo Fisher Scientific, USA).

### Production and purification of Armored RNA Particles (ARPs)

All inserts containing target sequences were subcloned into a linearized pET-MS2 vector, transformed, re-sequenced, and purified using the Plasmid Miniprep Kit (Zymo Research, USA). pET-MS2 vector contained the maturase and coat protein gene, and two packaging (pac) sites of MS2 bacteriophage. The targeted sequences were inserted between the two pac sites. Recombinant plasmids were transformed into *E. coli* BL21(DE3). Protein expression was induced with 1 mM IPTG (neoFroxx, Germany) at 37°C for 4 h. After the expression, RNA with target sequence was encapsulated in a protein coat. Cells were harvested by centrifugation, lysed (lysozyme treatment with freeze-thaw cycles), and digested with DNase I and RNase A (Zymo Research, USA). ARPs were purified from supernatants by CsCl (PanReac AppliChem, USA) density gradient centrifugation (200,000 ⅹ g, 22 h, 20°C) [35]. ARP fractions were collected and stored at 4°C. Primary fractions (3–4 per ARP variant) were screened using real-time RT-PCR with a mixed primer-probe system (Table 1). Optimal fractions were selected based on minimal Ct values: fraction 3 for MACV (S1 Fig in S1 File); fraction 4 for MACV_2 (S2 Fig in S1 File); and fraction 1 for MACV_10 (S3 Fig in S1 File) and MACV_14 (S4 Fig in S1 File).

### RNA purification and droplet digital PCR (ddPCR) analysis

ARPs underwent seven 10-fold serial dilutions (10^−1^–10^−7^) in ultrapure water. RNA was extracted from each ARP dilution using the RIBO-prep Kit (AmpliSens, Russia). Purified RNA was immediately analyzed after extraction. RNA quantification was performed using ddPCR (QX200™, Bio-Rad) with the One-Step RT-ddPCR Advanced Kit for Probes using flanking sequence-targeted primers/probes (SK_F/SK_R/SK_prb). Each reaction (20 μl) contained: 5 μl ddPCR Supermix; 2 μl reverse transcriptase; 1 μl 300 mM DTT; 1.8 μl each of 10 μM primers (SK_F, SK_R); 0.56 μl 10 μM SK_prb; 5.84 μl ultrapure water; and 2 μl RNA template. Reactions generated >10,000 accepted droplets, with thresholds manually set above no-template controls (NTC). Initial RNA concentrations were calculated by extrapolation (S5 Fig in S1 File). Undiluted sample and extreme dilutions (10^−1^, 10^−6^, 10^−7^) were excluded as outliers. The log_10_-linear correlation between copy number and dilution factor is shown in S5 Fig in S1 File.

### Real-Time RT-PCR

Optimized 25 μl reactions contained 12.5 μl of 2x RT-qPCR buffer (Biolabmix, Russia), 1 μl of 25x BioMaster mix (Biolabmix, Russia), 0.105 μl of each 100 μM primer (PCR_F, PCR_R, PCR_F.v2, PCR_R.v2), 0.075 μl of each 100 μM probe (PCR_prb, PCR_prb.v2), 0.93 µl DEPC-treated water (Biolabmix, Russia), and 10 μl RNA template. The aforementioned reaction mixture represents a primer/primer/probe concentration ratio of 7:7:5 per set. Alternative ratios (5:5:3 and 5:5:5) and annealing conditions (55/57/60°C for 20/25/30 s) were evaluated during optimization. The final thermocycling protocol on the CFX96 Touch (Bio-Rad, USA) was: 50°C for 15 min; 95°C for 5 min; and 40 cycles (95°C for 10 s, 60°C for 30 s) with HEX-channel detection.

### Real-Time RT-RPA assay

**Primer Screening**. Initial primer combinations were evaluated using the TwistAmp® Basic kit (TwistDx™, UK) under standard conditions. Amplification products were directly loaded onto 2% agarose TBE gels stained with 1% ethidium bromide (PanReac AppliChem, USA). Electrophoresis was performed at 80 V for 75 min (Mini-Sub Cell GT, Bio-Rad) alongside TriDye™ Ultra Low Range DNA Ladder (NEB, USA). Gels were imaged using a gelLITE Documentation System (Cleaver Scientific, UK).

**Optimized RT-exo RPA Protocol**. Lyophilized RPA master mixes were stored at –20°C. Reactions (50 μl) contained: 29.5 μl Primer Free Rehydration Buffer; 2.1 μl each of 10 μM forward/reverse primers; 0.6 μl of 10 μM fluorescent probe (THF-modified); 0.5 μl M-MuLV RT (200,000 U/ml, NEB, USA); 10.7 μl ultrapure water; 2.5 μl 280 mM magnesium acetate; and 2 μl RNA. After 40 seconds of vortexing, samples underwent isothermal amplification (40°C, 20 min) with minute-interval FAM-channel reads on a CFX96 Touch device (Bio-Rad, USA).

**Enzyme Variations**. For SuperScript™ IV RT (Invitrogen, 0.5 μl of 200 U/μl), RNase H (NEB, USA) was diluted in storage buffer to 0.5–2.5 U/μl. Storage buffer (pH 7.4) composition was: 50 mM KCl; 10 mM Tris-HCl; 0.1 mM EDTA; 1 mM DTT; 50% glycerol (PanReac AppliChem); and 200 μg/ml BSA (YACOO, China). Reactions used 2 μl RNA template and 1 μl RNase H, reducing water to 9.7 μl.

**Downscaled Screening & Portability**. Secondary screens used 25 μl reactions (all components halved). Field validation employed the Axxin T16-ISO device (Axxin, USA) with 30 minute amplification at 40°C. The device is battery-operated, lightweight (~2 kg), and can process 16 samples simultaneously with real-time fluorescence detection across three channels (FAM, HEX, ROX). The estimated market cost of approximately $5,000 - $7,000 USD represents a significant advantage compared to conventional real-time PCR systems such as the Bio-Rad CFX96 Touch ($20,000 - $30,000 USD), enhancing its suitability for resource-limited field settings. Notably, the stability of the lyophilized RPA master mix from −20°C to +25°C for ≥12 weeks supported its use for field deployment [36].

### Testing in serum samples

The RT-RPA and RT-PCR methods were evaluated using human serum samples spiked with ARPs. Serum samples were obtained from healthy donors following established ethical guidelines.

All experiments involving human serum were conducted in accordance with the principles of the Declaration of Helsinki and approved by the Institutional Review Board of the Saint Petersburg Pasteur Institute (St. Petersburg, Russia, No. 065−10, 05/07/2025). Informed consent was obtained from all participants prior to blood collection. All samples were fully anonymized prior to analysis.

### Cross-reactivity assessment

To evaluate analytical specificity, both RT-PCR and RT-RPA assays were tested against a panel of viruses from the strain collection of the Saint Petersburg Pasteur Institute. This panel had been previously validated using virus-specific RT-PCR assays in a prior study [37]. For the current evaluation, RNA and DNA extracts from these viruses were tested with the developed MACV-specific assays. The panel composition and corresponding commercial RT-PCR kits used for its initial validation are listed in Table 2. All commercial kits were used strictly according to the manufacturers’ instructions.

**Table 2:** Cross-reactivity assessment of MACV RT-PCR and RT-RPA assays against a panel of non-MACV viruses.

Viral species	Specific RT-PCR kit/ assay	Specific RT-PCR results	MACV Real-Time RT-PCR results	MACV Real-Time RT-RPA results
Lassa virus	LASV-Fl assay [37]	positive	negative	negative
Zaire ebolavirus	AmpliSens® FiloA-screen-FRT PCR kit (Russia) [38]	positive	negative	negative
Sudan ebolavirus		positive	negative	negative
Marburg virus		positive	negative	negative
Crimean-Congo hemorrhagic fever virus	AmpliSens® CCHFV-FRT PCR kit (Russia)	positive	negative	negative
Influenza A/H1N3	AmpliSens® Influenza virus A-type-FRT PCR kit (Russia)	positive	negative	negative
Influenza A/H3N2		positive	negative	negative
Influenza B	AmpliSens® Influenza virus A/B-FRT PCR kit (Russia)	positive	negative	negative
Yellow fever virus	AmpliSens® Yellow fever virus-FRT PCR kit (Russia)	positive	negative	negative
West Nile virus	AmpliSens® WNV-FRT PCR kit (Russia)	positive	negative	negative
Zika virus	AmpliSens® Zika virus -FRT PCR kit (Russia)	positive	negative	negative
Denge virus	AmpliSens® Dengue virus-FRT PCR kit (Russia)	positive	negative	negative
Tick borne encephalitis virus	AmpliSens® TBE-FRT PCR kit (Russia)	positive	negative	negative
Chikungunya virus	Chikungunya Detection kit (Real-Time Probe Based PCR) (HiMedia Laboratories Pvt Ltd., India)	positive	negative	negative
Kemerovo virus, strain 21/10	KEMV-Fl assay [39]	positive	negative	negative
Human Rotavirus A	AmpliSens® Rotavirus/ Norovirus/ Astrovirus-FRT PCR kit (Russia)	positive	negative	negative
Rabies virus	RABV-Fl assay [40]	positive	negative	negative
Human Cytomegalovirus 5	AmpliSens® CMV-FRT PCR kit (Russia)	positive	negative	negative
Human parvovirus B19	AmpliSens® Parvovirus B19-FRT PCR kit (Russia)	positive	negative	negative
Severe acute respiratory syndrome-related coronavirus-2	COVID-19Amp (St. Petersburg Pasteur Institute, Russia) [41]	positive	negative	negative

## Results

### Target sequence selection

All available *M. machupoense* (MACV) complete L segment sequences were aligned using Mega v.11 software (S6 Fig in S1 File). We chose a 136-nucleotide fragment of the RNA-dependent RNA polymerase gene as a target sequence. The reference sequence used was GenBank NC_005079.1 in the region from 1334 to 1469.

**Fig 1:**
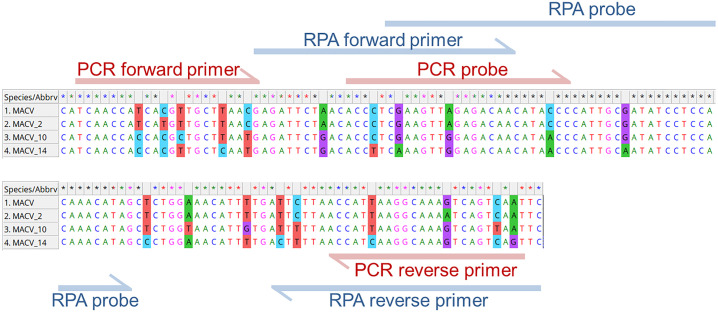
Sequence alignment of target variants. Nucleotide substitutions relative to MACV are highlighted. Binding sites for real-time RT-PCR and RT-RPA primers/probes are indicated.

This sequence is unique for MACV, but exhibits variability across genetic variants. This deliberately selected non-conserved region was chosen to demonstrate a solution to a common challenge in diagnosing highly variable viruses. The selected sequence is conserved in the Carvallo strain (AY619642.1, JN794583.1, KM198593.1, AY216511.2, MT015969.1) and several isolates (KU978786.1, KU978789.1). However, it contains one or more single-nucleotide polymorphisms (SNPs) in the Chicava strain (AY624354.1, KU978785.1), the Mallele strain (AY619644.1, JN794585.1), and other isolates (KU978790.1, KU978805.1, KU978784.1, KU978787.1, KU978791.1, KU978788.1). Consequently, the reference sequence (MACV) and three variant sequences with 2, 10, and 14 nucleotide substitutions were selected as model targets. These were designated as MACV_2, MACV_10, and MACV_14 (representing KU978805.1, AY619644.1, and KU978787.1, respectively). Their alignment is shown in Fig 1 with binding sites for PCR and RPA primers/probes highlighted.

### Real-Time RT-PCR assay

#### Real-Time PCR optimization.

Preliminary optimization was carried out using a plasmid containing the MACV sequence. Primers PCR_F, PCR_R, and probe PCR_prb were designed to avoid dimerization and selected based on lowest Ct values. Optimization of primer and probe concentrations showed that the best ratio (forward/reverse/probe) was 7:7:5, yielding optimal performance (S2 Table in S1 File). Annealing temperature and duration optimization identified 60°C for 30 seconds as optimal (S3 Table in S1 File). The final protocol is detailed in the Materials and Methods section.

This primer-probe set was tested on plasmids containing the variant sequences (MACV_2, MACV_10, MACV_14). As expected, increasing numbers of substitutions correlated with higher Ct values, reflecting reduced reaction efficiency (S7 Fig in S1 File). Amplification of MACV_14 was not detected.

To ensure detection of all variants, a second primer/probe set incorporating variant-matched substitutions (PCR_F.v2, PCR_R.v2, PCR_prb.v2) was introduced. Using a mixture of both primer sets, all control plasmids were successfully amplified (S8 Fig in S1 File).

#### Real-Time RT-PCR with ARPs.

**Fig 2:**
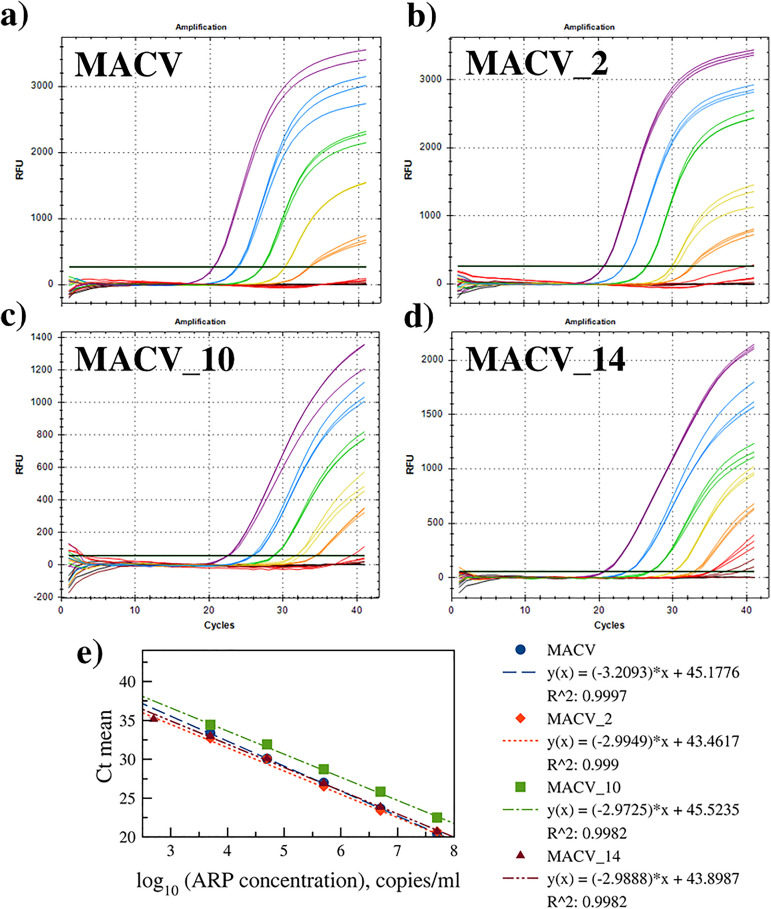
Real-time RT-PCR amplification plots using the mixed primer set on ARPs containing a) MACV, b) MACV_2, c) MACV_10, and d) MACV_14 target sequences. Serial 10-fold dilutions are shown (copies/ml): 5ⅹ10^1^ (brown), 5ⅹ10^2^ (red), 5ⅹ10^3^ (orange), 5ⅹ10^4^ (yellow), 5ⅹ10^5^ (green), 5ⅹ10^6^ (blue), 5ⅹ10^7^ (purple), and no template control (NTC, black). Panel e) shows standard curve with mean Ct values (± SD, n = 3) versus log_10_(ARP concentration).

The optimized assay was tested on RNA extracted from MACV-containing ARPs, previously quantified by ddPCR. Seven ten-fold serial dilutions (5 × 10^7^ to 5 × 10^1^ copies/ml) of each ARP variant (MACV, MACV_2, MACV_10, MACV_14) were analyzed. The assay achieved a limit of detection (LOD) of 5 × 10^3^ copies/ml (100% replicate positivity) for all variants (Fig 2). This equates to 100 target copies per reaction (10 copies/μl input RNA). As shown in Fig 2e, linear relationships between Ct value and log_10_(ARP concentration) were observed across the range 5 × 10^3^–5 × 10^7^ copies/ml (except 5 × 10^2^ for MACV_14).

### Real-Time RT-RPA assay

#### RPA primer and probe selection.

Recombinase polymerase amplification (RPA) efficiency critically depends on primer design. An initial screen of 2 forward (1F, 2F) and 3 reverse (4R, 5R, 6R) primers (6 combinations) assessed amplification by gel electrophoresis. The 1F/4R pair produced the highest-intensity amplicon band, suggesting superior efficiency (S9 Fig in S1 File).

**Fig 3:**
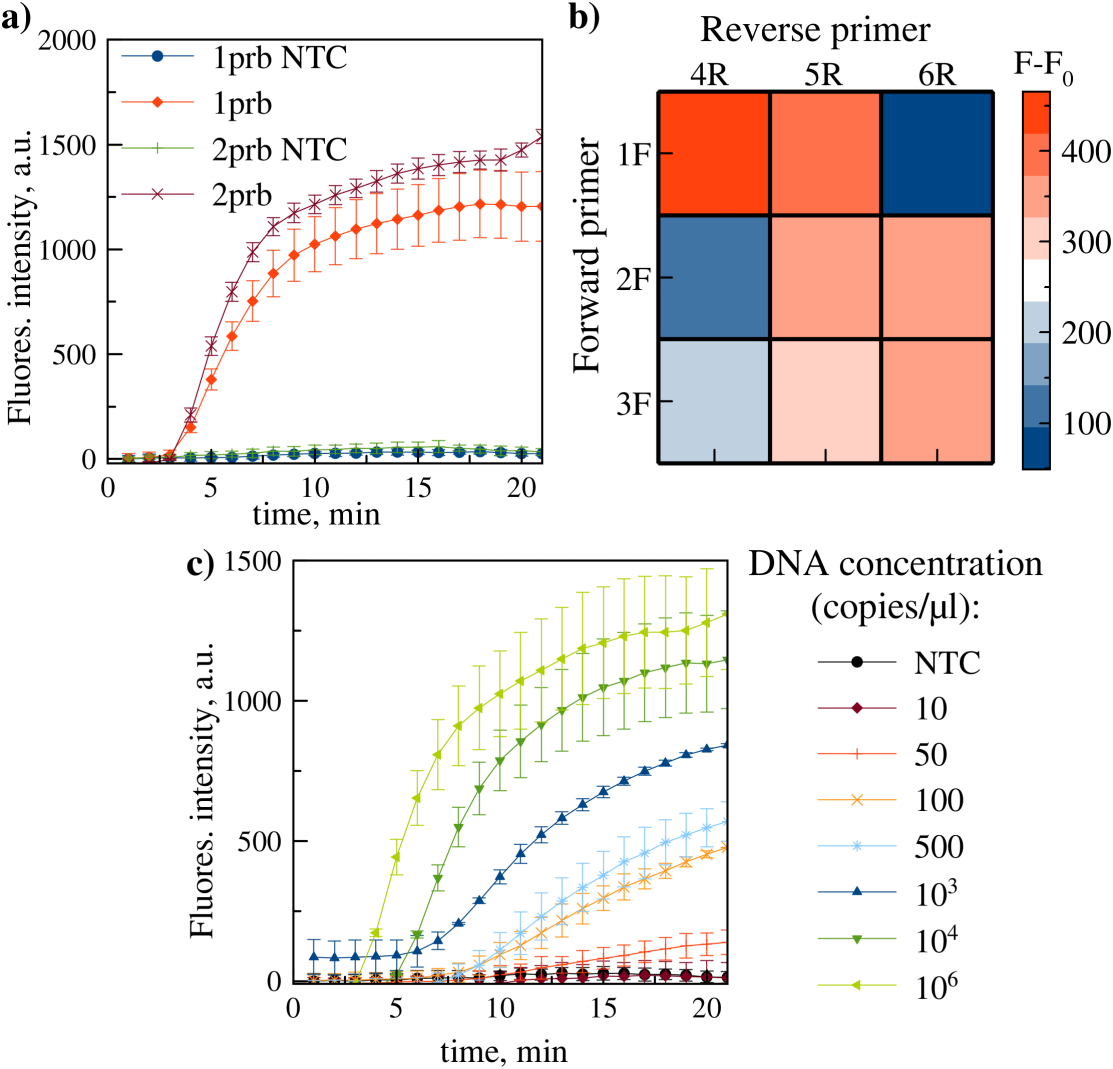
Real-time RPA assay. a) Real-time RPA kinetics comparing probes 1prb and 2prb. b) heatmap of fluorescence intensity (F-F0, background-subtracted) for primer pair screening (forward: 1F, 2F, 3F; reverse: 4R, 5R, 6R) with probe 2prb. c) real-time RPA kinetics using primer/probe set 1F/4R/2prb on serially diluted DNA template. Data represent mean ± SD (n = 2 replicate reactions).

This pair was used for real-time RPA development. Two exo probes (1prb, 2prb), each containing a tetrahydrofuran (THF) residue and complementary to opposite strands, were designed. Both probes feature the motif [dT-FAM]-[THF]-[dT-BHQ1], enabling initial FAM fluorescence quenching by BHQ1 via proximity. Cleavage at THF by exonuclease separates the fluorophore-quencher pair, thereby releasing signal. Comparative testing identified probe 2prb as yielding higher fluorescence (Fig 3a).

Primer/probe compatibility was further evaluated by screening the selected probe (2prb) with 3 forward (1F, 2F, 3F) and 3 reverse (4R, 5R, 6R) primers (9 combinations). Heatmap analysis confirmed 1F/4R as the optimal pair for 2prb (Fig 3b). Sensitivity testing of the 1F/4R/2prb set on DNA template dilutions established a LOD of 10–50 copies/reaction (Fig 3c).

#### Testing Real-Time RT-RPA with ARPs.

**Fig 4:**
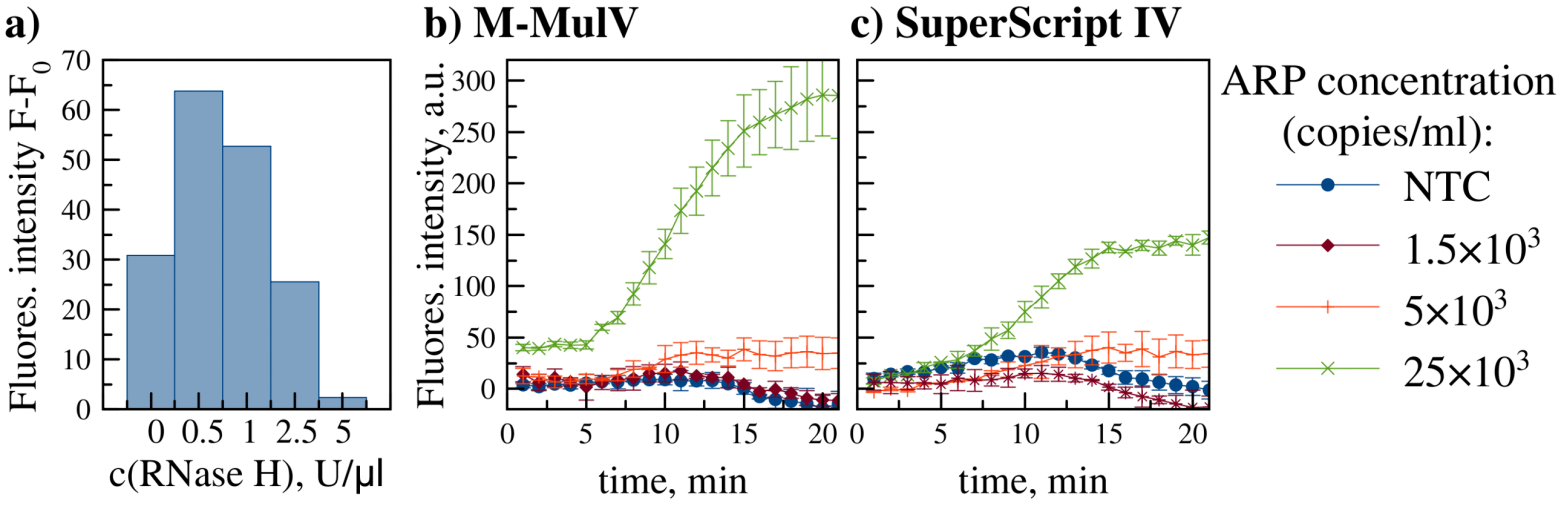
Real-time RT-RPA assay with ARPs. **a)** Relative fluorescence (F-F_0_) at endpoint (20 min) for RT-RPA assays using SuperScript IV RT supplemented with varying concentrations of RNase H (0 - 5 U/µl). b) real-time RT-RPA amplification kinetics using optimized conditions (M-MuLV RT or SuperScript IV RT + 0.5 U/µl RNase H) and ARPs (1.5 × 10^3^–2.5 × 10^4^ copies/ml). Data represent mean ± SD (n = 2).

Assay performance with RNA was evaluated using MACV-containing ARPs (target sequence encapsulated within MS2 capsid, mimicking viral RNA structure). Reverse transcriptase (RT) was incorporated into the RPA mix. M-MuLV and its engineered mutant, SuperScript IV (offering higher processivity and thermostability), were compared. As SuperScript IV lacks intrinsic RNase H activity, exogenous RNase H was added. The optimal RNase H concentration was determined by testing 1 µl additions at 0, 0.5, 1.0, 2.5, and 5.0 U/µl concentrations. As shown in Fig 4a, the 0.5 U/µl concentration yielded maximal amplification efficiency and was selected for subsequent experiments.

Both RT systems achieved comparable LODs (5 × 10^3^ ARP copies/ml) as shown in Fig 4. However, M-MuLV generated significantly higher fluorescence at 2.5 × 10^4^ copies/ml and was selected as the preferred transcriptase.

#### Optimization of RPA for different genetic variants.

**Fig 5:**
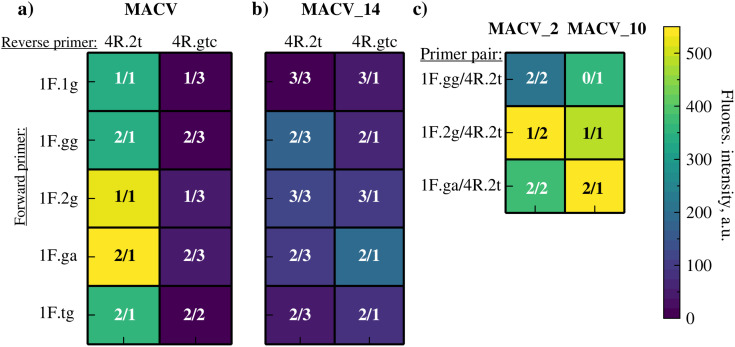
Heatmap of endpoint fluorescence intensity (F-F_0_) for redesigned primer pairs screened with probe 2prb across target variants. Primer variants: forward (1F.1g, 1F.gg, 1F.2g, 1F.ga, 1F.tg) and reverse (4R.2t, 4R.gtc). Numbers within cells (x/y) indicate substitutions in forward (x) and reverse (y) primers relative to original sequences. Panels: **a)** MACV, **b)** MACV_14, **c)** MACV_2 and MACV_10.

The 1F/4R/2prb set showed reduced efficiency against variants: ~ 40% lower fluorescence for MACV_2 and MACV_10; and no detection of MACV_14 (S10 Fig in S1 File). To ensure robust detection across all genetic variants, two strategies were pursued. The first approach focused on rational primer redesign to incorporate compensatory point substitutions. Ideally, these minimize mismatches, while seeking a single “compromise” primer pair effective against all variants. We systematically screened 5 modified forward primers (1F variants) and 2 modified reverse primers (4R variants). Each contained from 1 to 3 nucleotide substitutions relative to the original sequences. Combinatorial screening results across the target templates are presented as a heatmap map (Fig 5). Primer pair 1F.2g/4R.2t demonstrated the most balanced amplification performance and was selected as the optimal compromise candidate. However, testing this redesigned pair on ARP serial dilutions revealed a LOD of 5 × 10^4^ copies/ml (S11 Fig in S1 File). This represents a 10-fold reduction in sensitivity compared to the original set (1F/4R/2prb) with the MACV target.

The second strategy, analogous to multiplex PCR approaches, involved adding a second, variant-specific primer-probe set designed to perfectly match MACV_14, the sequence with the highest substitution burden. This set consisted of primer ‘1F.gtag’, primer ‘4R.gctc’, and probe ‘2prb.ga’. Initial attempts to simply add this second set to the reaction mixture (resulting in a doubling of the total primer concentration) led to significant reaction inhibition (S12a Fig in S1 File). This issue was resolved by halving the concentrations of each individual primer (both original and new sets) within the combined mixture. This adjustment successfully overcame inhibition, while maintaining amplification capability (S12b Fig in S1 File).

**Fig 6:**
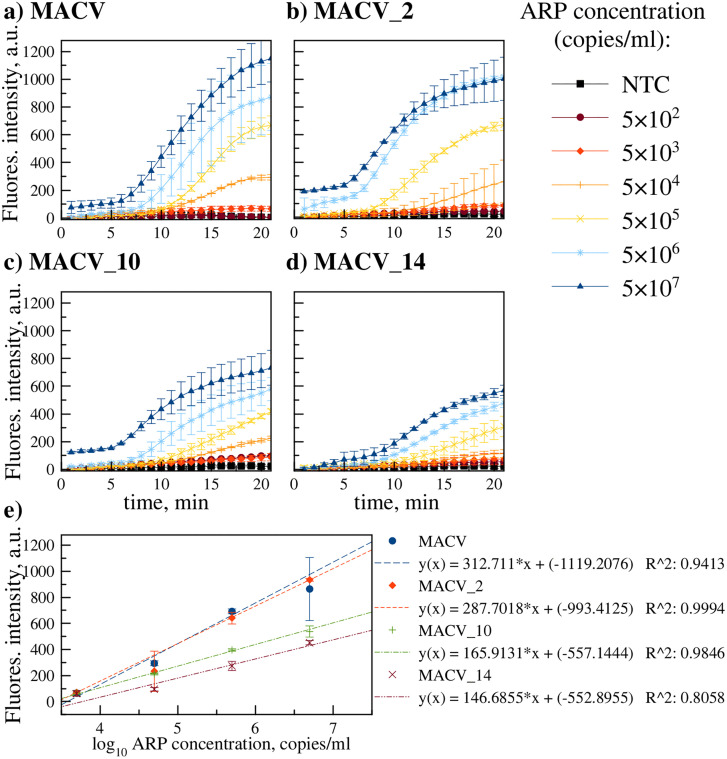
Real-time RT-RPA amplification kinetics with ARP serial dilutions containing: a) MACV, b) MACV_2, c) MACV_10, d) MACV_14. The optimized dual primer/probe set mixture was used (1F/4R/2prb + 1F.gtag/4R.gctc/2prb.ga, each at half concentration). LOD is indicated (5 × 10^3^ copies/ml). Panel e) linear dependence of fluorescence signal on log_10_(ARP concentration). Data represent mean ± SD (n = 2).

Testing the optimized dual primer/probe mixture on ARP serial dilutions demonstrated an improved LOD of 5 × 10^3^ copies/ml (Fig 6). This sensitivity matched the performance level previously achieved for real-time RT-RPA with the single primer set and was equivalent to the LOD obtained in the real-time RT-PCR assay.

Linear approximation in a specific concentration range (5 × 10^3^–5 × 10^6^ copies/ml) makes real-time RT-RPA (Fig 6e) semi-quantitative, similar to real-time RT-PCR. However, it should be noted that with increasing concentration, the endpoint fluorescence value no longer fits this line due to reaction saturation. The linearity range in real-time PCR does not have this problem (Fig 2e).

#### Application of Real-Time RT-RPA with a portable device using serum-spiked samples.

Field-deployable diagnostics are essential for neglected tropical diseases like Bolivian hemorrhagic fever. We evaluated the optimized real-time RT-RPA assay on the battery-operated, isothermal Axxin T16-ISO fluorimeter using MACV ARPs. While the LOD remained 5 × 10^3^ copies/ml, extending the run time to 30 minutes (from 20) was necessary to maintain sensitivity (S13 Fig in S1 File). Portable PCR systems, though available, were not assessed in this study.

**Fig 7:**
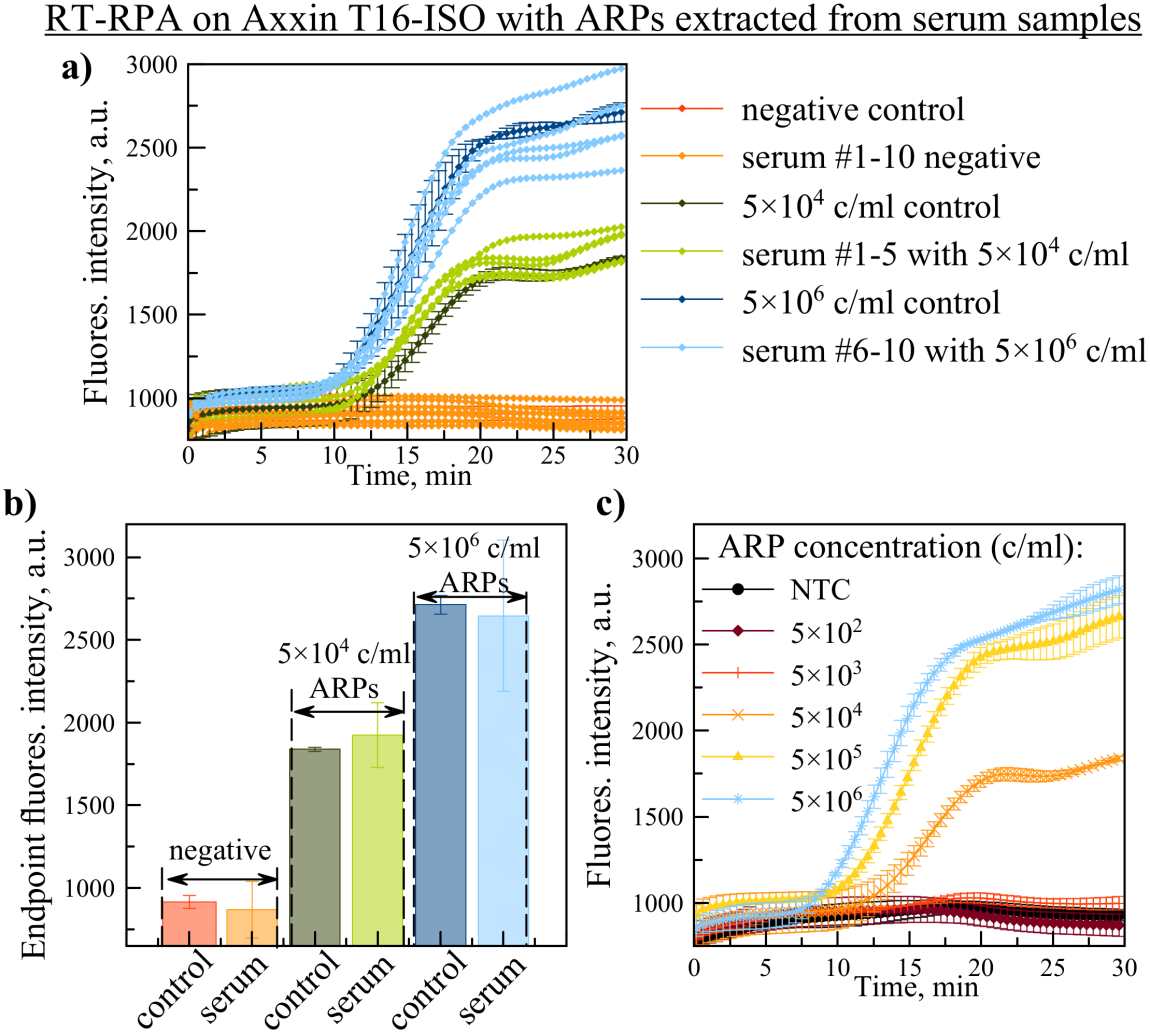
Evaluation of RT-RPA assay on an Axxin T16-ISO using serum-spiked samples. **a)** Amplification kinetics for MACV ARPs spiked into human serum and negative controls. Corresponding control samples with ARPs diluted in nuclease-free water are shown for comparison. **b)** Mean endpoint fluorescence for the samples shown in panel **(a)**. **c)** Determination of the assay’s LOD for ARPs spiked into serum, demonstrating a LOD of 5 × 10^3^ copies/ml. Data represent mean ±SD (n = 5 biological replicates for panel **(b)**; n = 2 reaction replicate for panel **(c)**).

To assess the assay’s robustness against potential inhibitors present in complex biological matrices, we tested the optimized RT-RPA system using MACV ARPs spiked into human serum. On the portable Axxin T16-ISO fluorimeter, RT-RPA successfully detected all positive samples (5/5 at 5 × 10^6^ copies/ml and 5/5 at 5 × 10^4^ copies/ml); while all negative controls (10/10 serum samples without ARPs) yielded no signal (Figs 7a and 7b). This resulted in 100% agreement, with no false-positive or false-negative results, indicating an absence of significant reaction inhibition or interference from serum components. The performance of serum samples was compared to ARPs diluted in nuclease-free water. Furthermore, the LOD of the real-time RT-RPA assay on the portable device was confirmed to be 5 × 10^3^ copies/ml for ARPs spiked into serum (Fig 7c).

For comparison, RT-PCR analysis of the serum-spiked ARP samples also showed 100% reliability for positive and negative results, with a mean Ct value variation of less than 1 cycle for replicates at the same concentration (S14 Fig, S4 Table in S1 File). The RT-PCR assay demonstrated a linear relationship between the mean Ct value and the logarithm of the ARP concentration (S15 Fig in S1 File).

### Analytical specificity testing: cross-reactivity assessment

To evaluate the potential for cross-reactivity and further validate the specificity of the developed assays, both the real-time RT-PCR and RT-RPA were tested against a comprehensive panel of non-MACV viruses from the strain collection of the Saint Petersburg Pasteur Institute. This panel, previously utilized and characterized in a study by Dedkov et al. [37], was designed to include viruses that could be relevant for differential diagnosis in endemic regions or pose a general biothreat. Crucially, it included the Lassa virus (LASV) from the same Arenaviridae family, providing a stringent test for specificity within the arenavirus group. The panel also encompassed other viral hemorrhagic fever agents (e.g., Ebola, CCHFV), arboviruses (e.g., Dengue, Zika), and respiratory pathogens.

All viral RNA samples in the panel were confirmed to be positive using specific commercial RT-PCR kits [37], as detailed in Table 2. When tested with our MACV-specific assays, both the real-time RT-PCR and RT-RPA yielded negative results for all 19 viruses in the panel, confirming no detectable cross-reactivity under the tested conditions. This provides strong empirical evidence for the high specificity of both diagnostic methods. It is important to note that while the panel included the Old World arenavirus LASV, other New World mammarenaviruses from South America were not available for testing in our collection.

## Discussion

The persistent threat of neglected tropical diseases, such as Machupo virus infection, demands urgent innovation in diagnostic tools, particularly for pathogens with pandemic potential. As a zoonotic arenavirus transmitted from rodents to humans, Machupo exemplifies high-risk agents where genetic variability, driven by cross-species adaptation, can rapidly enhance transmissibility and virulence. With fatality rates exceeding 25%, and no licensed vaccines, early detection remains the primary barrier against outbreaks. Our study directly addresses this need through parallel development of real-time RT-PCR and RT-RPA assays. Both were rigorously compared using identical armored RNA targets and detection parameters to ensure equitable performance evaluation. The results of the comparison are briefly presented in Table 3.

**Table 3:** Comparative analysis of the real-time RT-PCR and RT-RPA assays.

Parameter	Real-Time RT-PCR	Real-Time RT-RPA
LOD (ARP copies/ml)	5 × 10^3^	5 × 10^3^ (lab & portable)
LOD (RNA copies/reaction)	100	20
Time to result	1 h 30 min	20 min (lab), 30 min (portable)
Multiplex capability	yes (dual sets)	yes (dual sets)
Isothermal amplification	no	yes
Reaction cost (USD)†	1.32	6.15
Portable application	Palm PCR‡ (20 min, ~ $4.56)	Axxin T16-ISO (30 min)

† including primers/probes; ‡ theoretical estimate using the Palm PCR™ Express One-step qRT-PCR Kit (Ahram Biosystems) and a pack of special tubes. The latter: type IIA Palm PCR™ real-time sample tubes.

Both assays developed in our study achieved analytically relevant sensitivity (LOD 5 × 10^3^ ARP copies/ml), while demonstrating robust detection across genetically diverse MACV strains. When translated to identical units (target input), RT-PCR detected 100 RNA copies/reaction, while RT-RPA showed enhanced efficiency at 20 RNA copies/reaction, reflecting its superior tolerance to low-input samples. The real-time RT-PCR protocol, optimized with dual primer/probe sets, provides a cost-effective solution (~$1.32/reaction) for laboratories with existing infrastructure, delivering results in about 1 hour and 30 minutes. Critically, its multiplex design overcame challenges posed by highly variable targets (e.g., MACV_14 with 14 substitutions). In contrast, the RT-RPA assay, representing isothermal amplification, enabled field deployment, though at higher reagent costs (~$6.15/reaction). When assessed on the Axxin T16-ISO portable device, RT-RPA maintained its 5 × 10^3^ copies/ml sensitivity with only a 10 minute time extension (30 minutes total runtime).

While portable PCR systems like the Palm PCR™ could theoretically reduce RT-PCR runtime to 20 minutes at ~$4.56/reaction, our study experimentally confirms RT-RPA’s superior field adaptability. The Axxin-validated RT-RPA workflow eliminates thermal cycling dependencies and reduces power requirements; these are critical advantages in resource-limited settings.

To contextualize the performance of our assays, we compared them to an existing RT-qPCR assay for MACV, which reported a similar LOD of 5 copies/µl [4], which is equivalent to the 5 × 10^3^ copies/ml LOD established for our assays. This demonstrates that our optimized RT-RPA and RT-PCR assays deliver state-of-the-art analytical sensitivity. However, our study provides a significant advancement in model rigor. While the earlier validation relied on synthetic RNA, our evaluation utilized armored RNA particles. ARPs more accurately simulate genuine viral particles due to the presence of a protective MS2 bacteriophage capsid shell, and our protocol incorporated a nucleic acid extraction step from these particles. This approach subjects the assays to a more challenging and biologically relevant environment, strengthening the confidence in the reported performance metrics and their closer approximation to real-world application.

Despite these strengths, our study has limitations. The primary constraint is the use of armored RNA particles in a controlled laboratory setting and spiked clinical matrix, rather than true clinical specimens from infected hosts. It is important to note that the testing with spiked serum represents a model system and does not replace comprehensive clinical trials. Nevertheless, these experiments serve as a critical step in validation, providing a robust demonstration that the developed real-time RT-RPA system maintains its sensitivity and specificity following nucleic acid extraction from a complex biological fluid. The absence of inhibition and the preservation of the detection limit underscore the assay’s robustness and its potential for reliable detection in future clinical studies. While this approach provided a safe and standardized model for initial validation, future studies must assess assay performance on a well-characterized panel of human and animal samples to definitively establish clinical sensitivity and specificity. Furthermore, such evaluation will require the development and integration of robust sample preparation protocols, including effective viral inactivation steps for safe handling and efficient nucleic acid extraction methods optimized for complex clinical matrices such as blood or tissue homogenates. Moreover, for true field-deployment, future work will focus on integrating the RT-RPA assay with simplified, equipment-free sample processing methods for nucleic acid extraction, to create a complete sample-to-answer diagnostic solution. The comparative evaluation of different extraction methods for field use represents an important direction for further research.

Beyond conventional PCR and isothermal amplification (e.g., real-time RPA), emerging diagnostics leverage CRISPR-Cas systems, often coupled with pre-amplification (e.g., RPA) for sensitivity [42]. In our parallel study targeting the identical MACV fragment, we developed a RT-RPA/DETECTR assay (Cas12a) [43]. While CRISPR-based methods offer potential for multiplexing and exceptional specificity, our implementation encountered challenges in achieving a simple homogeneous format, requiring spatial separation of RPA and Cas12a reagents to prevent inhibition. This combined assay achieved an LOD of 5 × 10^4^ copies/ml with a 60 min total runtime. In contrast, the homogeneous real-time RT-RPA system presented here, which requires no post-amplification handling, demonstrated a tenfold higher sensitivity and simpler workflow. This comparison, based on our specific development experience, underscores a common trade-off in assay design between maximum sensitivity, procedural simplicity, and the potential for additional functionality. The optimal choice depends heavily on the intended use case and available infrastructure.

Collectively, these studies provide the first direct comparison of PCR, RPA, and RPA-CRISPR platforms for arenavirus detection. Three key insights emerged. First, simplicity favors real-time RPA because single-tube, single-step protocols minimize operational errors in field settings. It is important to note, however, that the higher reagent cost of RPA must be weighed against the operational advantages of speed and portability for each specific use case. Second, speed-sensitivity trade-offs show that CRISPR-enhanced methods add complexity, without sensitivity gains, versus optimized RPA. Third, real-time RPA satisfies urgent frontline screening, while PCR remains for confirmatory testing. This methodological hierarchy aligns with the WHO’s recommendations for decentralized diagnostics: rapid frontline triage (RPA), followed by centralized verification (PCR).

## Conclusion

This study successfully solves an important diagnostic problem in Machupo virus detection by developing and evaluating two complementary molecular approaches adapted to different healthcare settings. The real-time RT-PCR assay delivers laboratory-grade sensitivity (5 × 10^3^ ARP copies/ml, 100 RNA copies/reaction) at minimal cost, while utilizing multiple primer-probe sets to overcome viral genetic diversity. Meanwhile, the real-time RT-RPA platform achieves equivalent analytical sensitivity under tested conditions in 20–30 minutes and can be potentially applied in the field using portable devices, although at higher reagent cost.

RPA’s operational flexibility, combined with equivalent sensitivity to gold-standard PCR, positions it as a transformative tool for frontline Machupo surveillance. Future efforts should focus on reducing RT-RPA reagent costs and evaluating these assays against clinical samples in endemic regions. The balance between cost-effectiveness and field-adaptability will be crucial for implementation. Accessible diagnostics are critical for timely control of zoonotic viral outbreaks, especially in dynamic settings featuring evolutionary changes.

## Supporting information

S1 File Contains all supporting figures and tables (S1 Table. Complete list of oligonucleotide sequences with modifications. S1 Fig. Fraction selection for MACV armored RNA particles (ARPs) by real-time RT-PCR. Fluorescence kinetics for fractions 1 (blue), 2 (green), and 3 (purple). Optimal fraction (3) was selected based on lowest Ct value. S2 Fig. Fraction selection for MACV_2 armored RNA particles (ARPs) by real-time RT-PCR. Fluorescence kinetics for fractions 1 (blue), 2 (green), 3 (purple), and 4 (orange). Optimal fraction (4) was selected. S3 Fig. Fraction selection for MACV_10 armored RNA particles (ARPs) by real-time RT-PCR. Fluorescence kinetics for fractions 1 (blue), 2 (green), 3 (purple), and 4 (orange). Optimal fraction (1) was selected. S4 Fig. Fraction selection for MACV_14 armored RNA particles (ARPs) by real-time RT-PCR. Fluorescence kinetics for fractions 1 (blue), 2 (green), 3 (purple), and 4 (orange). Optimal fraction (1) was selected. S5 Fig. Linear fit analysis of ARP quantification by ddPCR. Log_10_-transformed concentrations of serial ARP dilutions (MACV) versus ddPCR-calculated RNA copies/ml. Outliers (undiluted, 10^-1^, 10^-2^, 10^-7^ dilutions) excluded. S6 Fig. Sequence alignment of Machupo virus L segment fragments. GenBank accession numbers: NC_005079.1 (reference), MT015969.1, KU978805.1, KU978791.1, KU978790.1, KU978789.1, KU978788.1, KU978787.1, KU978786.1, KU978785.1, KU978784.1, KM198593.1, JN794585.1, JN794583.1, AY624354.1, AY619644.1, AY619642.1, AY358021.2, AY216511.2. S2 Table. Primer/probe concentration optimization for real-time RT-PCR. Mean Ct values (Bio-Rad CFX Maestro) for MACV detection using varying concentrations of PCR_F, PCR_R, and PCR_prb. The optimal concentration ratio was 7:7:5. S3 Table. Annealing parameter optimization for real-time RT-PCR. Mean Ct values (Bio-Rad CFX Maestro) testing annealing temperatures (55–60°C) and durations (20–30 s). Optimal conditions: 60°C for 30 s. S7 Fig. Real-time RT-PCR amplification for variant plasmids (initial primer set). Targets: MACV (blue), MACV_2 (green), MACV_10 (purple), MACV_14 (black), NTC (orange). Threshold: 0.05 (red). Note: MACV_14 was undetected. S8 Fig. Real-time RT-PCR with dual primer/probe sets. Successful detection of all variants: MACV (blue), MACV_2 (green), MACV_10 (purple), MACV_14 (black). Note: NTC (orange), auto-threshold (dark green). S9 Fig. RPA primer screening by gel electrophoresis. Lanes 1–6: amplicons from every primer pair (1F/4R, 1F/5R, 1F/6R; 2F/4R, 2F/5R, 2F/6R) with positive control. NTC 1–6: negative controls. Red highlight: RPA product from optimal 1F/4R pair (136 bp). Ladder: 10–700 bp. S10 Fig. Endpoint fluorescence of RT-RPA with MACV variants. Normalized signal for the 1F/4R/2prb set shows reduced detection of MACV_2/MACV_10 and failure to detect MACV_14. S11 Fig. RT-RPA sensitivity for redesigned primers (1F.2g/4R.2t/2prb). Serial ARP dilutions of (a) MACV, (b) MACV_2, (c) MACV_10, (d) MACV_14. LOD: 5 × 10⁴ copies/ml. Mean ± SD, n = 2. S12 Fig. Dual primer/probe concentration optimization. Amplification kinetics with (a) full-concentration mix (inhibition) vs (b) half-concentration mix (optimal) for all variants. S13 Fig. Portable real-time RT-RPA validation on the Axxin T16-ISO device. Amplification kinetics for MACV ARPs using the optimized dual primer/probe mixture. S14 Fig. RT-PCR assay with ARPs spiked and extracted from serum samples. 10 negative serum samples spiked with water (orange), 1 NTC (black), 10 positive serum samples spiked with ARPs (blue), 1 positive control ARPs sample spiked in water (green). All samples are made in triplicate. S4 Table. Mean Ct value for RT-PCR assay with ARPs spiked and extracted from serum samples. S15 Fig. Linear fit for mean Ct values (± SD, n = 3) versus log_10_(ARP concentration) for real-time RT-PCR on ARPs containing serum samples.).
(PDF)

S1 Data Raw data for Figs 2–7, Table 2. (XLSX)s

S2 Data Raw data for Figs S1-S5, S7, S8. (XLSX)

S3 Data Raw data for Figs S10-S15. (XLSX)

S4 Data Raw data for Tables S2-S4. (XLSX)

S2 File Gel image for Fig S9. (PDF)
