# Supplementary material for: Isothermal real-time RT-RPA for Machupo virus detection: Field-adaptable sensitivity comparable with laboratory PCR
Source: PLoS One. 2026 Jan 12;21(1):e0340488. doi: 10.1371/journal.pone.0340488 (PMC12795347; doi:10.1371/journal.pone.0340488)
Supplement: S1 File — Complete list of oligonucleotide sequences with modifications. S1 Fig. Fraction selection for MACV armored RNA particles (ARPs) by real-time RT-PCR. Fluorescence kinetics for fractions 1 (blue), 2 (green), and 3 (purple). Optimal fraction (3) was selected based on lowest Ct value. S2 Fig. Fraction selection for MACV_2 armored RNA particles (ARPs) by real-time RT-PCR. Fluorescence kinetics for fractions 1 (blue), 2 (green), 3 (purple), and 4 (orange). Optimal fraction (4) was selected. S3 Fig. Fraction selection for MACV_10 armored RNA particles (ARPs) by real-time RT-PCR. Fluorescence kinetics for fractions 1 (blue), 2 (green), 3 (purple), and 4 (orange). Optimal fraction (1) was selected. S4 Fig. Fraction selection for MACV_14 armored RNA particles (ARPs) by real-time RT-PCR. Fluorescence kinetics for fractions 1 (blue), 2 (green), 3 (purple), and 4 (orange). Optimal fraction (1) was selected. S5 Fig. Linear fit analysis of ARP quantification by ddPCR. Log10-transformed concentrations of serial ARP dilutions (MACV) versus ddPCR-calculated RNA copies/ml. Outliers (undiluted, 10-1, 10-2, 10-7 dilutions) excluded. S6 Fig. Sequence alignment of Machupo virus L segment fragments. GenBank accession numbers: NC_005079.1 (reference), MT015969.1, KU978805.1, KU978791.1, KU978790.1, KU978789.1, KU978788.1, KU978787.1, KU978786.1, KU978785.1, KU978784.1, KM198593.1, JN794585.1, JN794583.1, AY624354.1, AY619644.1, AY619642.1, AY358021.2, AY216511.2. S2 Table. Primer/probe concentration optimization for real-time RT-PCR. Mean Ct values (Bio-Rad CFX Maestro) for MACV detection using varying concentrations of PCR_F, PCR_R, and PCR_prb. The optimal concentration ratio was 7:7:5. S3 Table. Annealing parameter optimization for real-time RT-PCR. Mean Ct values (Bio-Rad CFX Maestro) testing annealing temperatures (55–60°C) and durations (20–30 s). Optimal conditions: 60°C for 30 s. S7 Fig. Real-time RT-PCR amplification for variant plasmids (initial primer set). Targets: MACV (blue), [file pone.0340488.s001.pdf]

Supporting information

Isothermal real-time RT-RPA for Machupo virus detection: field-adaptable sensitivity comparable with laboratory PCR

Marina A. Kapitonova[1]*, Anna V. Shabalina[1], Igor S. Sukhikh[1,#a], Artemiy A. Volkov[1,#b], Vladimir G. Dedkov[1,2], and Anna S. Dolgova[1]

[1]Laboratory of Pathogen Molecular Genetics, Saint Petersburg Pasteur Institute, St. Petersburg 197101, Russia
[2]Martsinovsky Institute of Medical Parasitology, Tropical and Vector Borne Diseases, First Moscow State Medical University (Sechenov University), Moscow 119048, Russia

[#a]Current Address: School of Biology and Environmental Science, University College Dublin, Belfield, Dublin 4 D04 V1W8, Ireland
[#b]Current Address: Faculty of Chemistry and Pharmacy, Institute for Biochemistry, University Freiburg, Freiburg 79104, Germany

***Correspondence**: kapitonova@pasteurorg.ru; kapitonova.marin@gmail.com

S1 Table. Complete list of oligonucleotide sequences with modifications.

Name	Sequence (5`→3`)
M_1	CATCAACCATCACGTTGCTTAACG
M_2	TGGGGTATGTTGTCTCTAACTTCGAGGGTGTTAGAATCTCGTTAAGCAACGTGATGGTTG
M_3	GAAGTTAGAGACAACATACCCCATTGCGATATCCTCCACAAACATAGCTCTGGAAACATT
M_4	GAATTGACTGACTTTGCCTTAATGGTTAAGAATCAAAATGTTTCCAGAGCTATGTTTGT
M_2_1	TCAACCATCATGTTGCTTAACGA
M_2_2	GAATTGACTGATTTTGCCTTAATGG
M_10_1	CATCAACCACCACGCTGC
M_10_2	GGTTATGTTGTCTCCAACTTCGAGGGTGTCAGAATCTCATTAAGCAGCGTGGTGGTTGAT
M_10_3	CGAAGTTGGAGACAACATAACCCATTGCGATATCCTCCACAAACATAGCTCTGGTAACAT
M_10_4	GAATTAACTGACTTTGCCTTAATGGTTAAAAATCACAATGTTACCAGAGCTATGTTTGTG
M_14_1	CATCAACCACCACGTTGC
M_14_2	GTTATGTTGTCTCCAACTTTGAAGGTGTCAGAATCTCATTGAGCAACGTGGTGGTTGATG
M_14_3	CTTCAAAGTTGGAGACAACATAACCCATTGCAATATCCTCCACAAACATAGCCCTGGAAA
M_14_4	GAACTGACTGACTTTGCCTTGATGGTTAAAAGTCAAAATGTTTCCAGGGCTATGTTTGTG
SK_F	AGTTGGAGGACATCAAGCAGCCATGCAAAT
SK_R	TGCTATGTCAGTTCCCCTTGGTTCTCT
SK_prb	[VIC] GAGACCATCAATGAGGAAGCTGCAGAATGG [BHQ1]
PCR_F	TCAACCATCAYGTTGCTTAACG
PCR_F.v2	TCAACCACCACGTTGCTCAATG
PCR_R	ATTGACTGAYTTTGCCTTAATGGT
PCR_R.v2	ACTGACTGACTTTGCCTTGATGGT
PCR_prb	[R6G] ACCCTCGAAGTTAGAGACAACATACCGAG [BHQ1]
PCR_prb.v2	[R6G] ACCTTCAAAGTTGGAGACAACATAACGAG [BHQ1]

RPA_1F	GAGATTCTAACACCCTCGAAGTTAGAGACAAC
RPA_2F	CGTTGCTTAACGAGATTCTAACACCCTCGAAG
RPA_3F	ATCACGTTGCTTAACGAGATTCTAACACCCTCG
RPA_1F.1g	GAGATTCTGACACCCTCGAAGTTAGAGACAAC
RPA_1F.gg	GAGATTCTGACACCCTCGAAGTTGGAGACAAC
RPA_1F.2g	GAGATTCTAACACCCTCGAAGTTGGAGACAAC
RPA_1F.ga	GAGATTCTGACACCCTCAAAGTTAGAGACAAC
RPA_1F.tg	GAGATTCTAACACCTTCGAAGTTGGAGACAAC
RPA_1F.gtag	GAGATTCTGACACCTTCAAAGTTGGAGACAAC
RPA_4R	GAATTGACTGACTTTGCCTTAATGGTTAAGAAT
RPA 5R	GACTGACTTTGCCTTAATGGTTAAGAATCAAA
RPA 6R	TGACTTTGCCTTAATGGTTAAGAATCAAAATGT
RPA_4R.2t	GAATTGACTGACTTTGCCTTAATGGTTAAAAAT
RPA_4R.gtc	GAACTGACTGACTTTGCCTTAATGGTTAAAAGT
RPA_4R.gctc	GAACTGACTGACTTTGCCTTGATGGTTAAAAGT
RPA_1prb	TAAGAATCAAAATGTTTCCAGAGCTATGTT [dT-FAM] [THF] [dT-BHQ1] GGAGGATATCGCAAT [Spacer C3]
RPA_2prb	CGAAGTTAGAGACAACATACCCCATTGCGA [dT-FAM] [THF] [dT-BHQ1] CCTCCACAAACATAG [Spacer C3]
RPA_2prb.ga	CGAAGTTGGAGACAACATAACCCATTGCGA [dT-FAM] [THF] [dT-BHQ1] CCTCCACAAACATAG [Spacer C3]

Key: standard bases (A,G,T,C); degenerate nucleotides (Y=C/T). Modifications: fluorophores R6G (rhodamine 6G), dT-FAM (fluorescein-dT), VIC; quenchers BHQ1 (Black Hole Quencher 1), dT-BHQ1 (Black Hole Quencher 1-dT); spacers THF (tetrahydrofuran spacer), spacer C3.

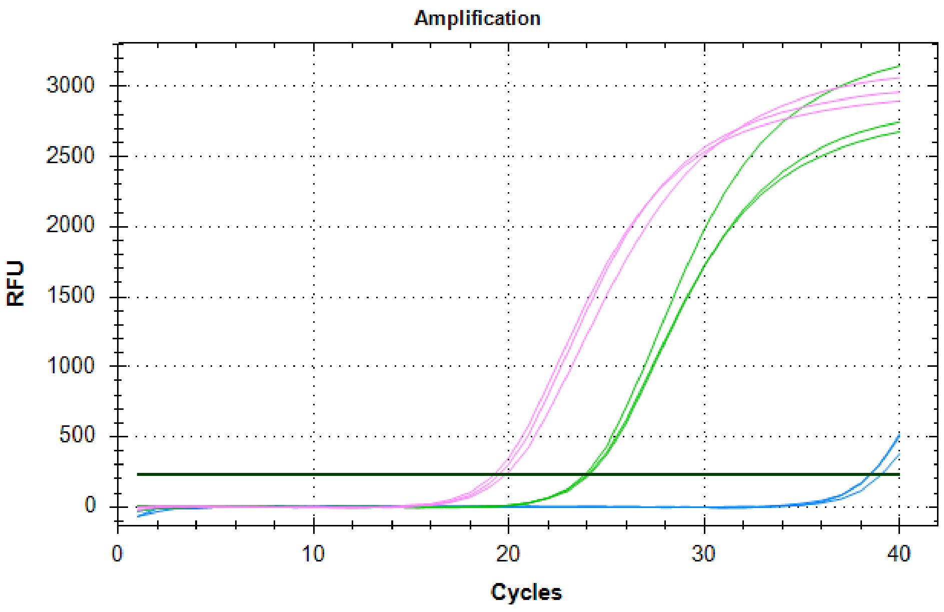

S1 Fig. Fraction selection for MACV armored RNA particles (ARPs) by real-time RT-PCR. Fluorescence kinetics for fractions 1 (blue), 2 (green), and 3 (purple). Optimal fraction (3) was selected based on lowest Ct value.

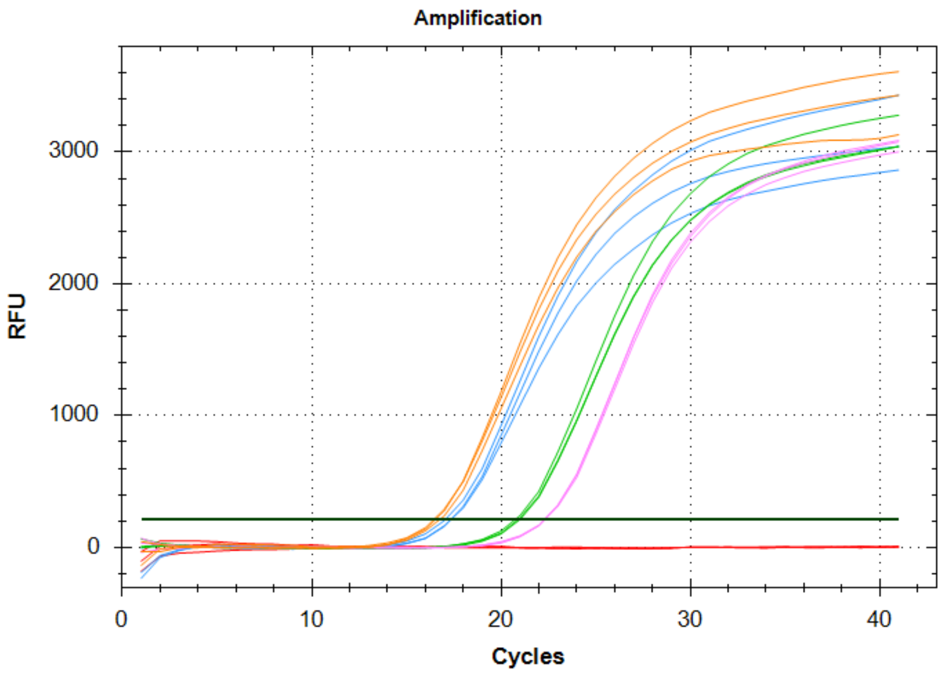

S2 Fig. Fraction selection for MACV_2 armored RNA particles (ARPs) by real-time RT-PCR. Fluorescence kinetics for fractions 1 (blue), 2 (green), 3 (purple), and 4 (orange). Optimal fraction (4) was selected.

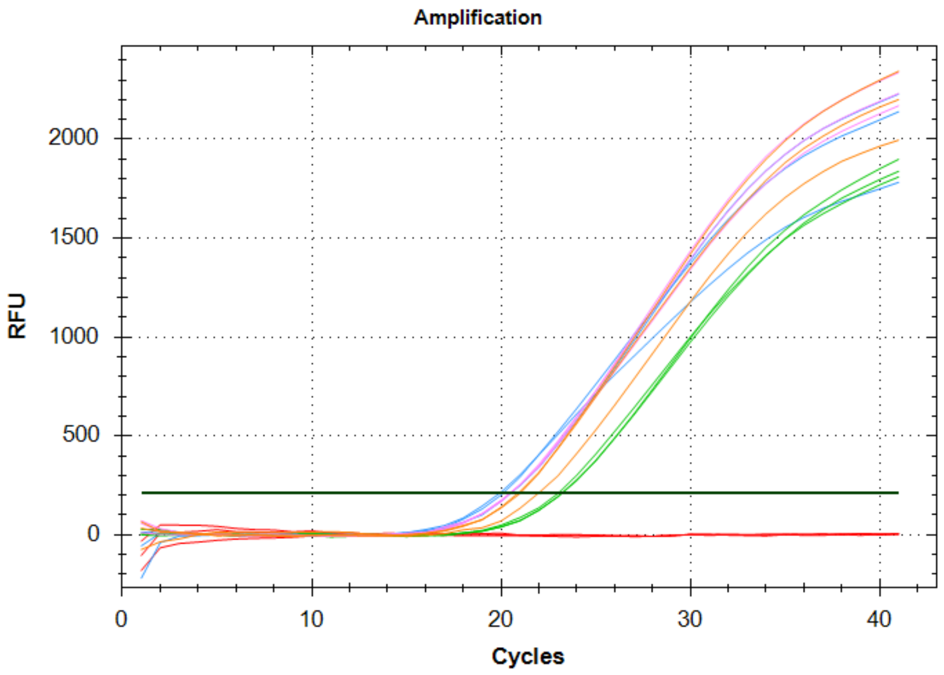

S3 Fig. Fraction selection for MACV_10 armored RNA particles (ARPs) by real-time RT-PCR. Fluorescence kinetics for fractions 1 (blue), 2 (green), 3 (purple), and 4 (orange). Optimal fraction (1) was selected.

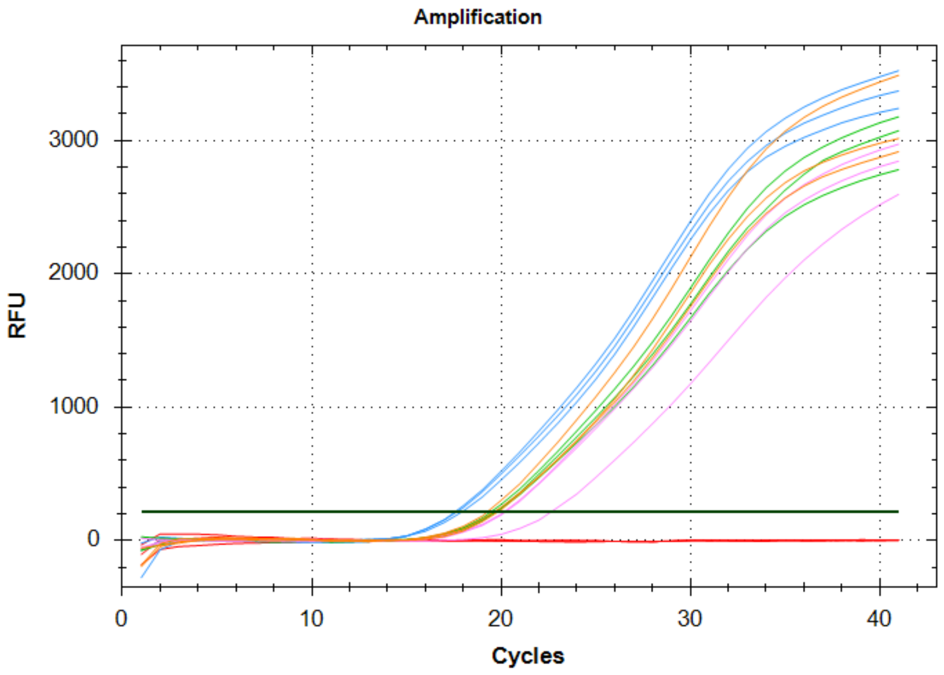

S4 Fig. Fraction selection for MACV_14 armored RNA particles (ARPs) by real-time RT-PCR. Fluorescence kinetics for fractions 1 (blue), 2 (green), 3 (purple), and 4 (orange). Optimal fraction (1) was selected.

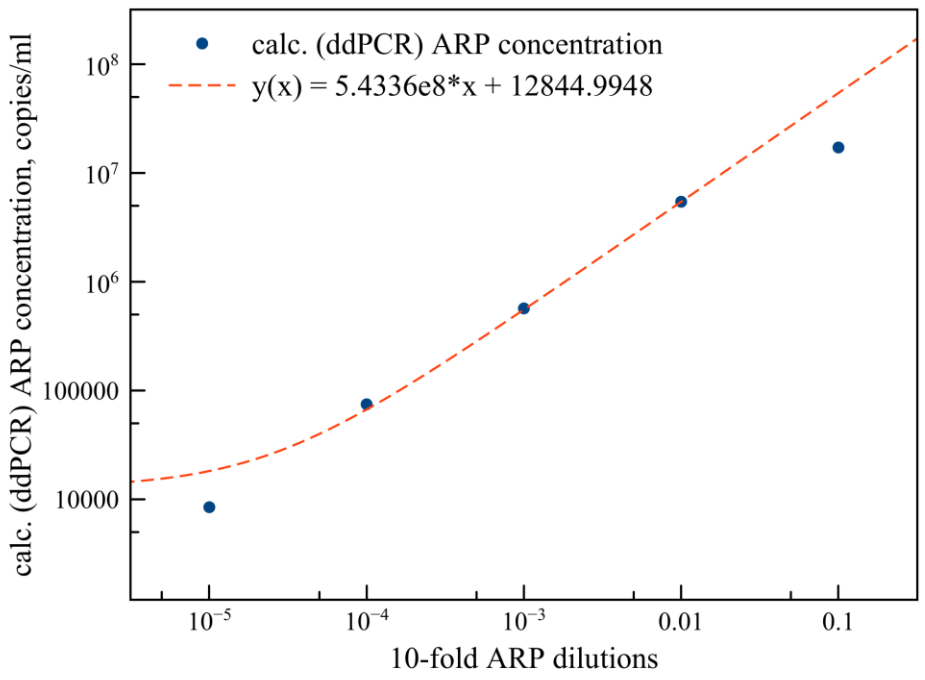

S5 Fig. Linear fit analysis of ARP quantification by ddPCR. Log_{10}-transformed concentrations of serial ARP dilutions (MACV) versus ddPCR-calculated RNA copies/ml. Outliers (undiluted, 10^{-1}, 10^{-6}, 10^{-7} dilutions) excluded.

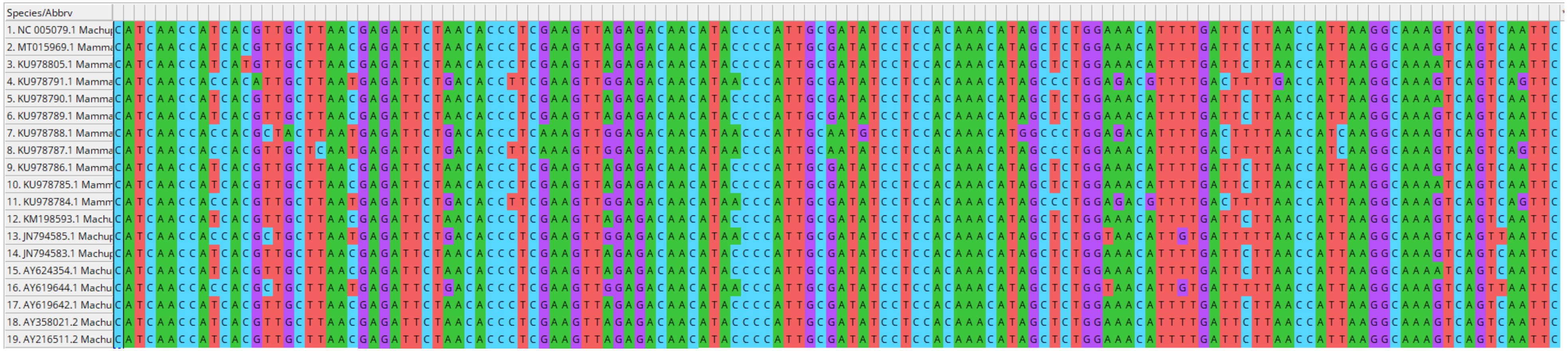

S6 Fig. Sequence alignment of Machupo virus L segment fragments. GenBank accession numbers: NC_005079.1 (reference), MT015969.1, KU978805.1, KU978791.1, KU978790.1, KU978789.1, KU978788.1, KU978787.1, KU978786.1, KU978785.1, KU978784.1, KM198593.1, JN794585.1, JN794583.1, AY624354.1, AY619644.1, AY619642.1, AY358021.2, AY216511.2.

S2 Table. Primer/probe concentration optimization for real-time RT-PCR. Mean Ct values (Bio-Rad CFX Maestro) for MACV detection using varying concentrations of PCR_F, PCR_R, and PCR_prb. The optimal concentration ratio was 7:7:5.

[PCR_F]:[PCR_R]:[PCR_prb] Concentration ratio	Ct mean
7:7:5	24.96
5:5:5	25.14
5:5:3	25.7

S3 Table. Annealing parameter optimization for real-time RT-PCR. Mean Ct values (Bio-Rad CFX Maestro) testing annealing temperatures (55-60℃) and durations (20-30 s). Optimal conditions: 60℃ for 30 s.

Time / Temperature	55℃	57℃	60℃
20 s	26.71	26.59	26.35
25 s	26.23	24.81	25.76
30 s	24.66	24.97	24.18

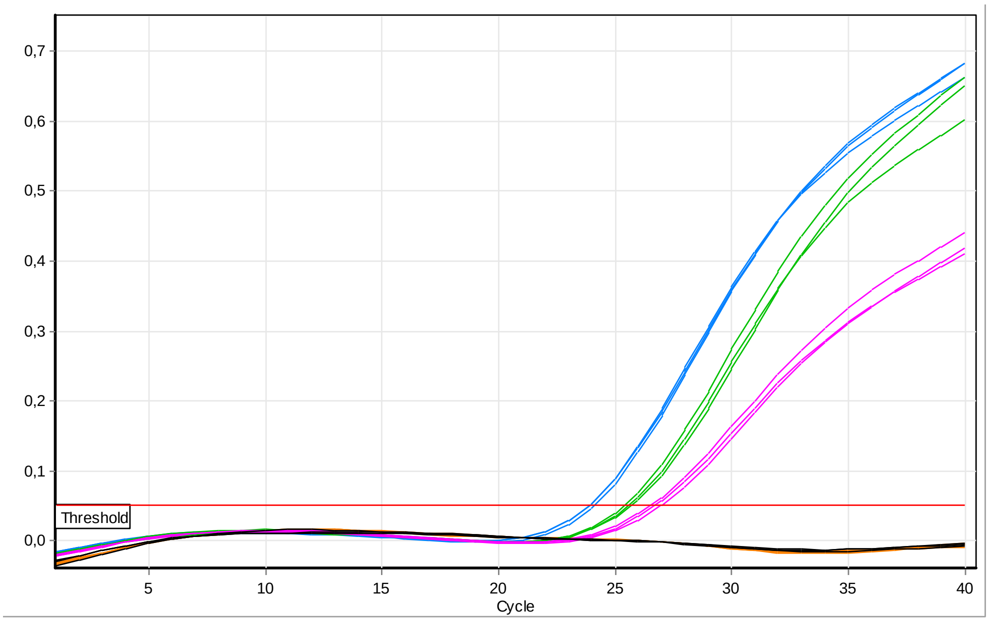

S7 Fig. Real-time RT-PCR amplification for variant plasmids (initial primer set). Targets: MACV (blue), MACV_2 (green), MACV_10 (purple), MACV_14 (black), NTC (orange). Threshold: 0.05 (red). Note: MACV_14 was undetected.

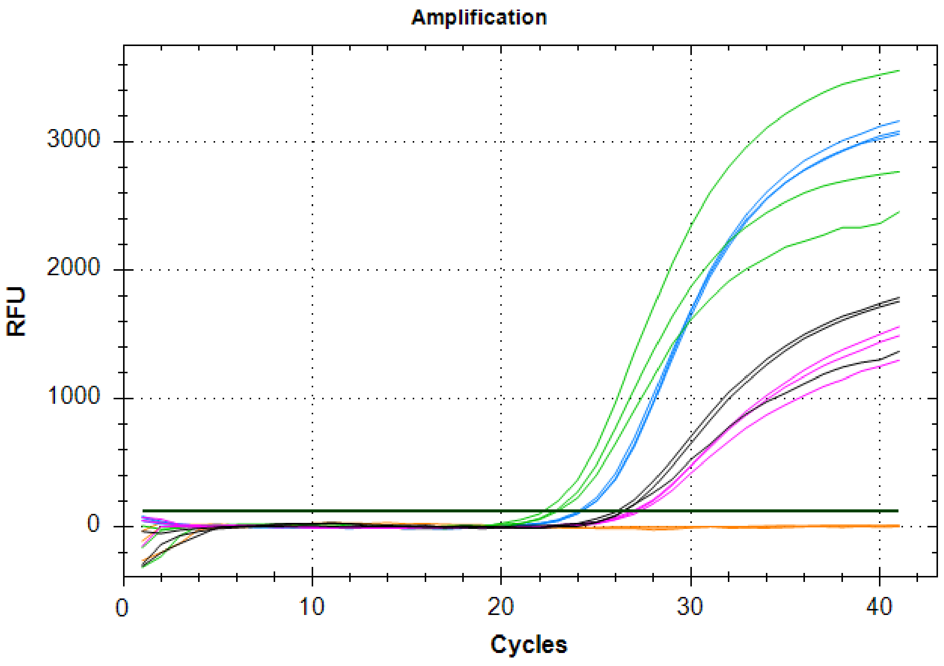

S8 Fig. Real-time RT-PCR with dual primer/probe sets. Successful detection of all variants: MACV (blue), MACV_2 (green), MACV_10 (purple), MACV_14 (black). Note: NTC (orange), auto-threshold (dark green).

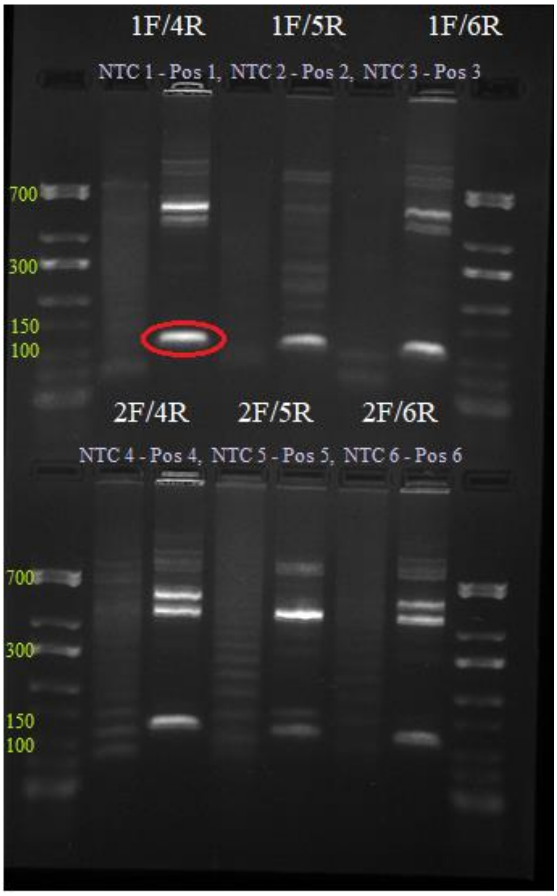

S9 Fig. RPA primer screening by gel electrophoresis. Lanes 1-6: amplicons from every primer pair (1F/4R, 1F/5R, 1F/6R; 2F/4R, 2F/5R, 2F/6R) with positive control. NTC 1-6: negative controls. Red highlight: RPA product from optimal 1F/4R pair (136 bp). Ladder: 10-700 bp.

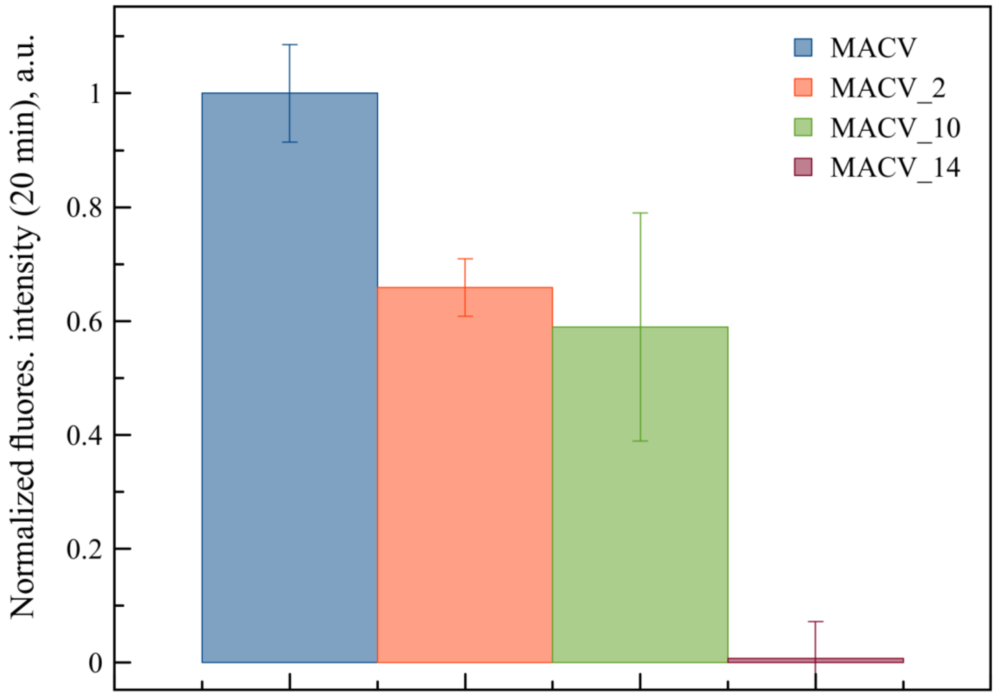

S10 Fig. **Endpoint fluorescence of RT-RPA with MACV variants.** Normalized signal for the 1F/4R/2prb set shows reduced detection of MACV_2/MACV_10 and failure to detect MACV_14.

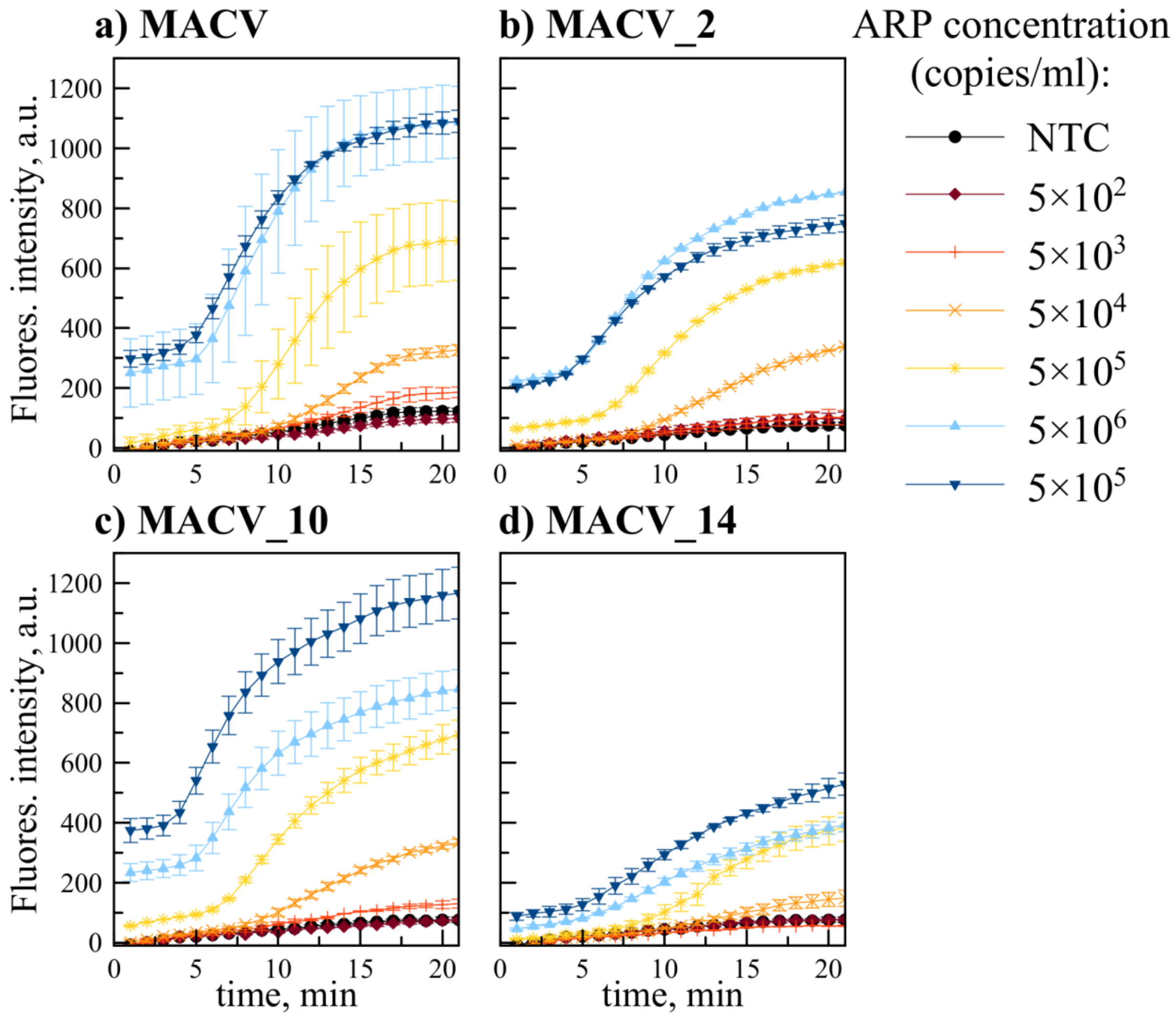

S11 Fig. RT-RPA sensitivity for redesigned primers (1F.2g/4R.2t/2prb). Serial ARP dilutions of (a) MACV, (b) MACV_2, (c) MACV_10, (d) MACV_14. LOD: 5×10^4 copies/ml. Mean ± SD, n=2.

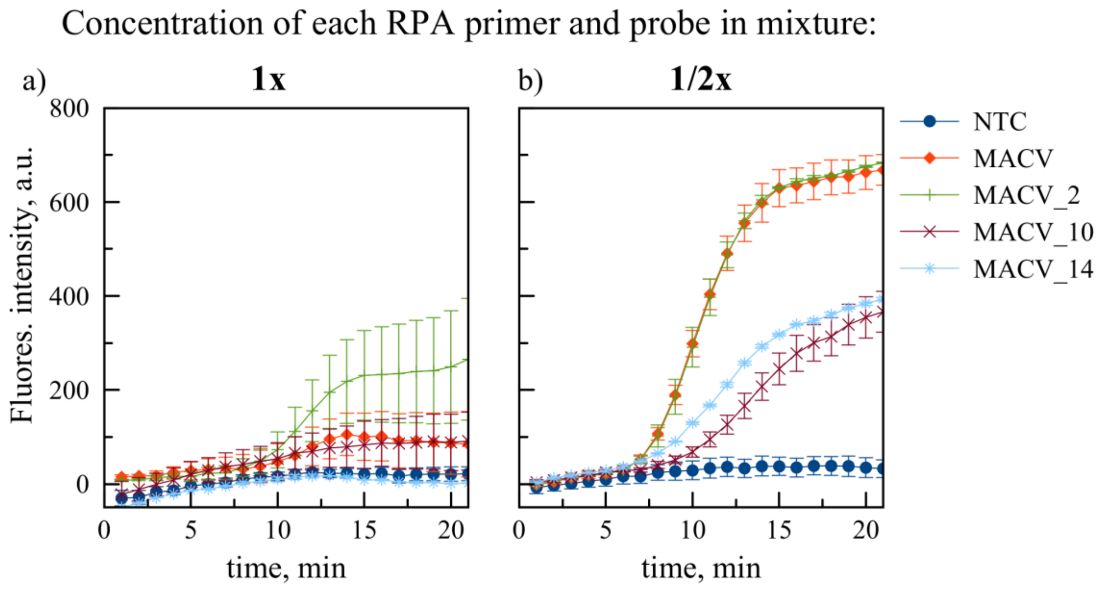

S12 Fig. **Dual primer/probe concentration optimization.** Amplification kinetics with (a) full-concentration mix (inhibition) vs (b) half-concentration mix (optimal) for all variants.

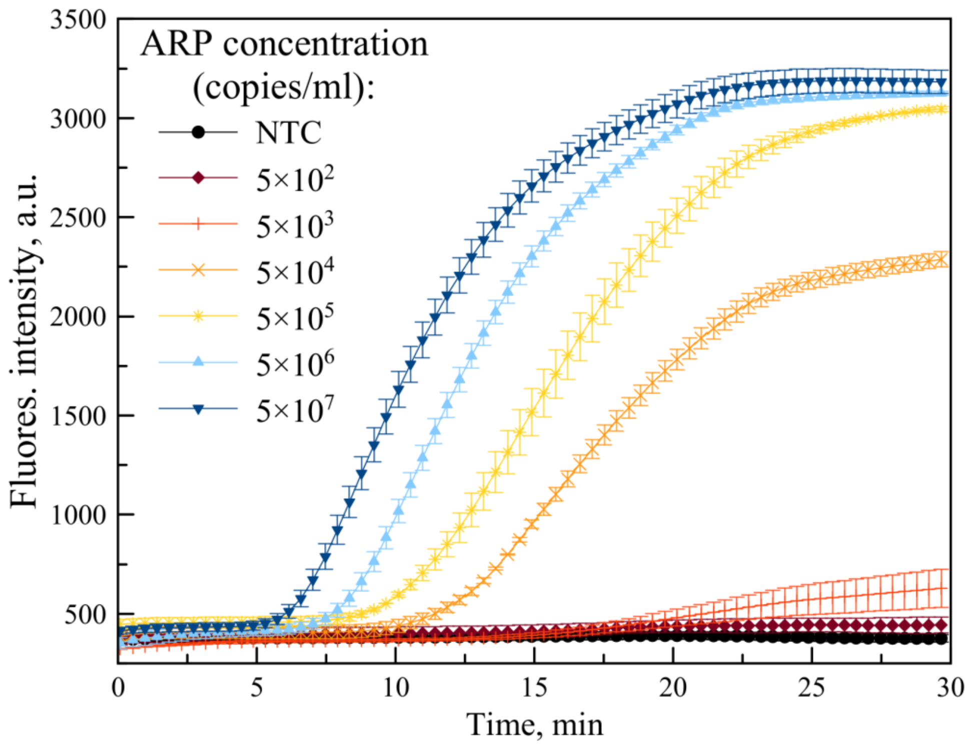

S13 Fig. Portable real-time RT-RPA validation on the Axxin T16-ISO device. Amplification kinetics for MACV ARPs using the optimized dual primer/probe mixture.

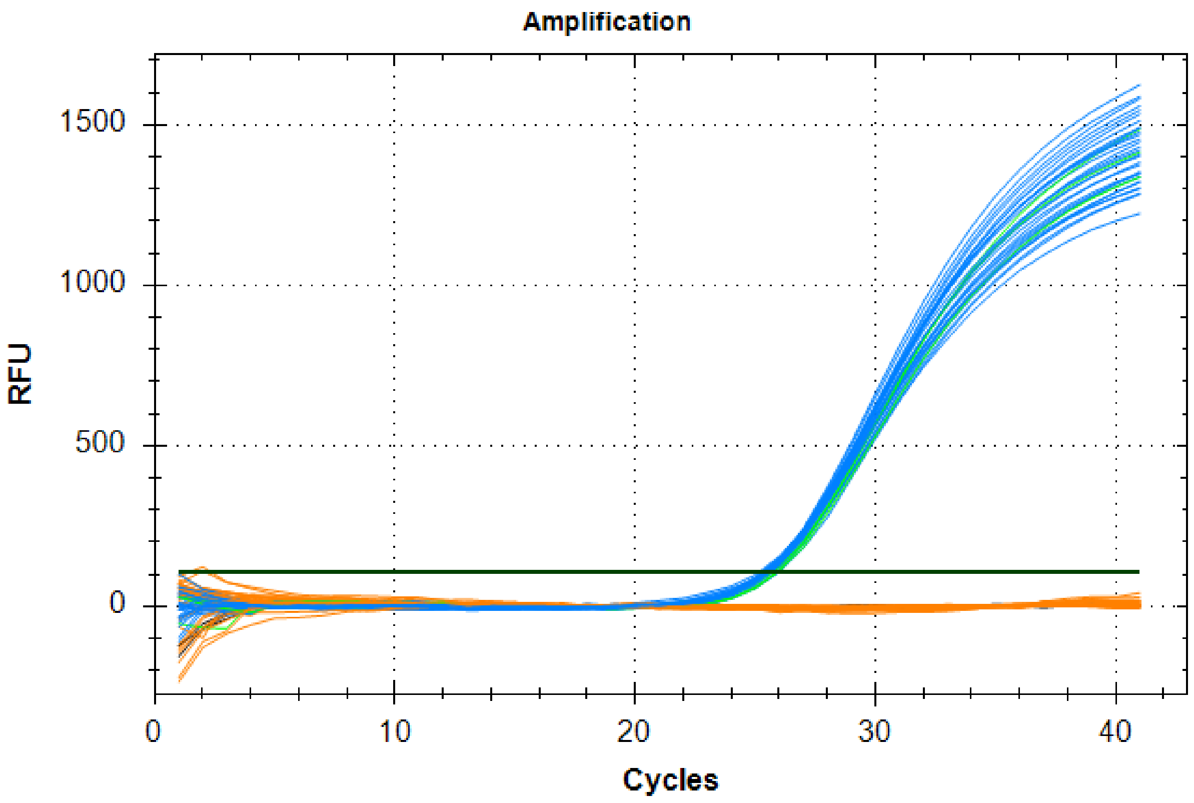

S14 Fig. RT-PCR assay with ARPs spiked and extracted from serum samples. 10 negative serum samples spiked with water (orange), 1 NTC (black), 10 positive serum samples spiked with ARPs (blue), 1 positive control ARPs sample spiked in water (green). All samples are made in triplicate.

S4 Table. Mean Ct value for RT-PCR assay with ARPs spiked and extracted from serum samples.

Sample type	Sample number	Ct mean (for 3 replicates)	Ct Std. Dev.
NTC	1	N/A	-
Negative serum sample	1	N/A	-
	2	N/A	-
	3	N/A	-
	4	N/A	-
	5	N/A	-
	6	N/A	-
	7	N/A	-
	8	N/A	-

	9	N/A	-
	10	N/A	-
Positive control from water	1	25.87	0.125
Positive serum samples	1	25.56	0.161
	2	25.49	0.130
	3	25.51	0.169
	4	25.58	0.415
	5	25.55	0.065
	6	25.49	0.173
	7	25.65	0.286
	8	25.45	0.153
	9	25.62	0.140
	10	25.52	0.156

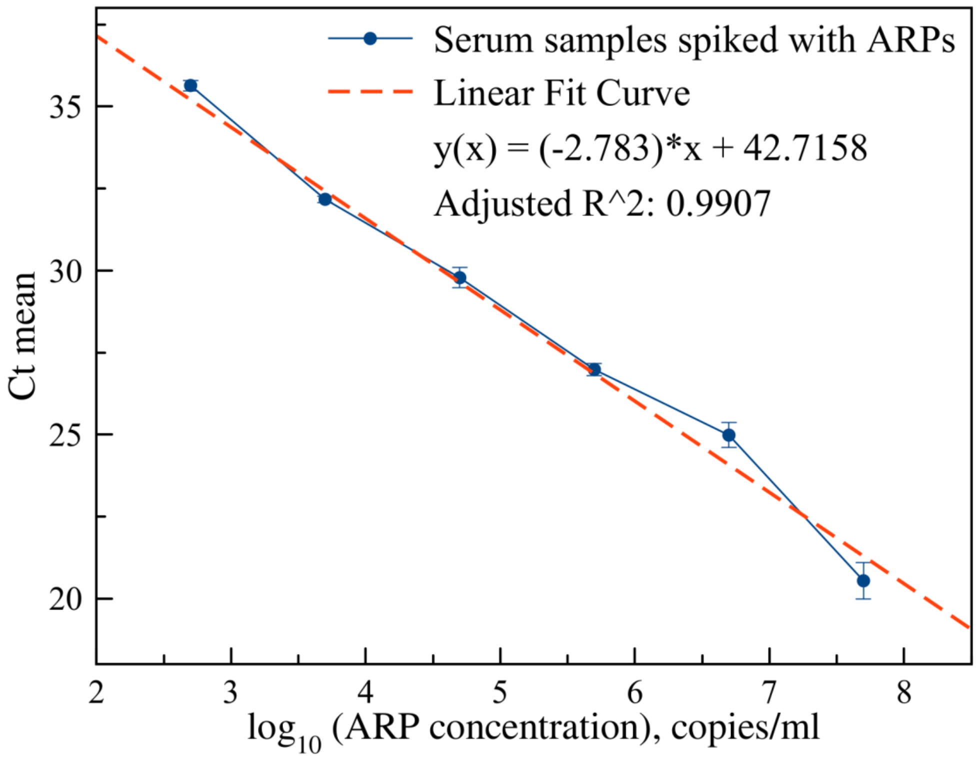

S15 Fig. Linear fit for mean Ct values (± SD, n=3) versus log_{10}(ARP concentration) for real-time RT-PCR on ARPs containing serum samples.
