# Supplementary figures and images for: Isothermal real-time RT-RPA for Machupo virus detection: Field-adaptable sensitivity comparable with laboratory PCR
Source: PLoS One. 2026 Jan 12;21(1):e0340488. doi: 10.1371/journal.pone.0340488 (PMC12795347; doi:10.1371/journal.pone.0340488)

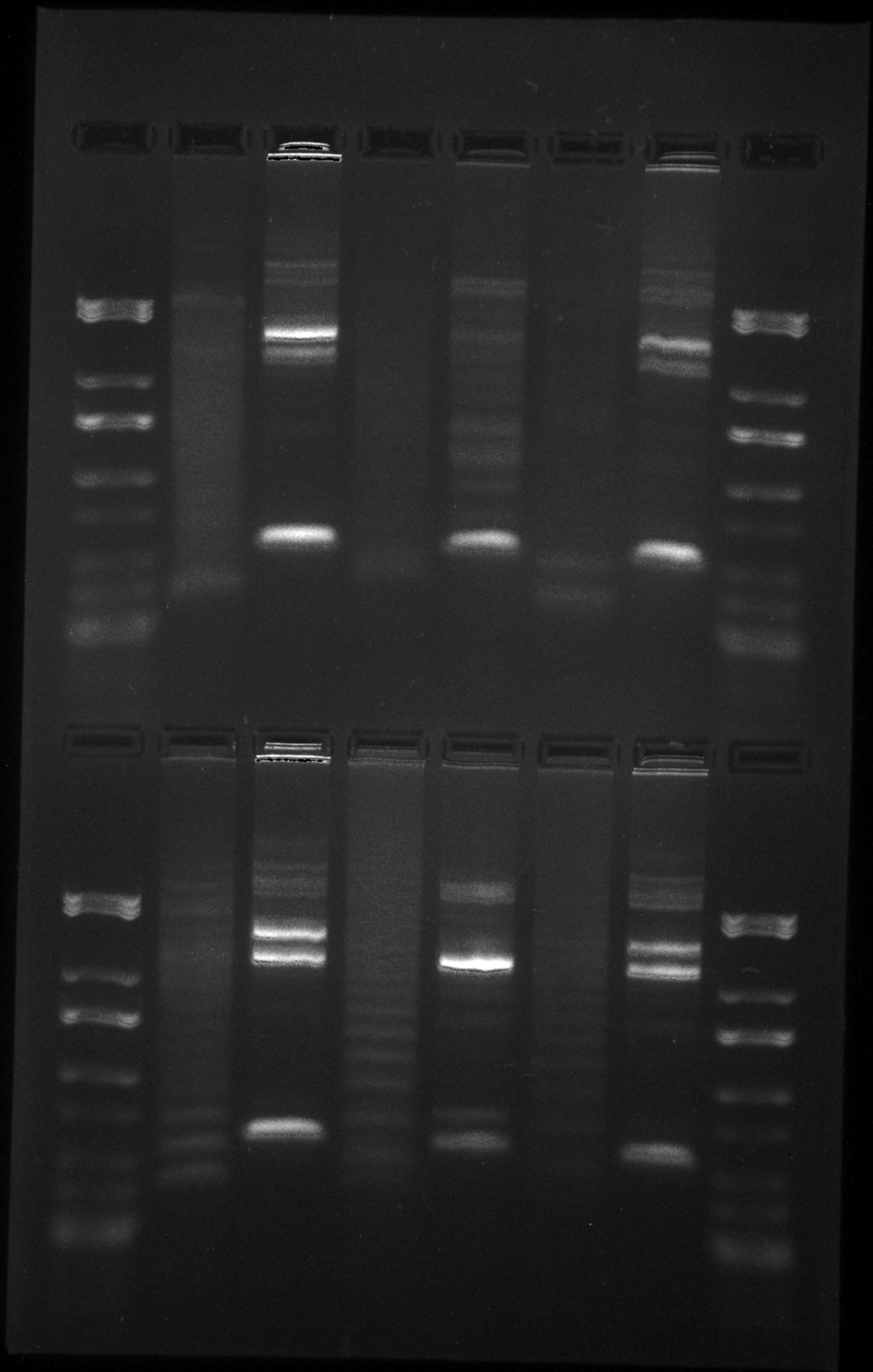

Supplement: S2 File — (PDF) [file pone.0340488.s006.pdf]
